# Integrated microbiome, metabolome, and proteome analysis identifies a novel interplay among commensal bacteria, metabolites and candidate targets in non‐small cell lung cancer

**DOI:** 10.1002/ctm2.947

**Published:** 2022-06-23

**Authors:** Xiang Qian, Hong‐Yan Zhang, Qing‐Lin Li, Guan‐Jun Ma, Zhuo Chen, Xu‐Ming Ji, Chang‐Yu Li, Ai‐qin Zhang

**Affiliations:** ^1^ The Cancer Hospital of the University of Chinese Academy of Sciences (Zhejiang Cancer Hospital) Hangzhou People's Republic of China; ^2^ Zhejiang Provincial Key Laboratory of Thoracic Tumor Hangzhou People's Republic of China; ^3^ Department of Comprehensive Ward Affiliated Hangzhou Cancer Hospital Zhejiang University School of Medicine Hangzhou People's Republic of China; ^4^ Zhejiang Chinese Medicine University Hangzhou People's Republic of China

**Keywords:** all‐trans‐retinoic acid, gut microbiota, lung cancer, metabonomics, nervonic acid, proteomics

## Abstract

**Background:**

Accumulation of evidence suggests that the gut microbiome, its specific metabolites, and differentially expressed proteins (DEPs) are related to non‐small cell lung cancer (NSCLC) pathogenesis. We now report the influences of the gut microbiota, metabolites, and DEPs on the mediation of NSCLC's chronic inflammation and immune dysregulation.

**Methods:**

We conducted 16S ribosomal RNA sequencing for the gut microbiome in healthy volunteers and NSCLC patients. Liquid chromatography–mass spectrometry (LC–MS) analysis was employed to explore differences between metabolites and DEPs in serum samples. Additionally, LC–MS‐based metabolomic analysis was conducted in 40 NSCLC tissues and 40 adjacent tissues. The omics data were separately analysed and integrated by using Spearman's correlation coefficient. Then, faecal microbiota transplantation (FMT) assay was used to assess the effects of the gut microbiome and specific metabolites in mice.

**Results:**

Faecal microbiome analysis revealed gut microflora dysbiosis in NSCLC patients with *Prevotella*, *Gemmiger*, and *Roseburia* significantly upregulated at the genus level. Then, we identified that nervonic acid/all‐trans‐retinoic acid level was negatively related to *Prevotella*. Additionally, a total of core 8 DEPs were selected in the proteome analysis, which mainly participated in the production of IL‐8 and NF‐κB pathways. CRP, LBP, and CD14 were identified as potential biomarkers for NSCLC. Transplantation of faecal microbiota from patients with NSCLC or *Prevotella copri*‐colonized recipient in mice resulted in inflammation and immune dysregulation. In turn, nervonic acid/all‐trans‐retinoic acid treatment improved the phenotype of C57BL/6 mice bearing *P. copri*‐treated Lewis lung cancer (LLC).

**Conclusions:**

Overall, these results pointed out that *P. copri*‐nervonic acid/all‐trans‐retinoic acid axis may contribute to the pathogenesis of NSCLC.

## Graphical abstract

- *Prevotella*, *Gemmiger*, and *Roseburia* were significantly upregulated at the genus level in NSCLC patients.
- Nervonic acid/all‐trans‐retinoic acid was negatively related to *Prevotella*.
- CRP, LBP, and CD14 were identified as potential biomarkers for NSCLC.
- Transplantation of faecal microbiota from patients with NSCLC or *Prevotella copri*‐colonized recipient mice caused inflammation and immune dysregulation in Lewis lung cancer (LLC) cells‐bearing C57BL/6 mice.
- Nervonic acid/all‐trans‐retinoic acid improved the *Prevotella copri*‐treated LLC cells‐bearing C57BL/6 mice phenotype.

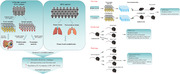

## 1 BACKGROUND

Lung cancer (LC) is one of the commonest fatal malignancies in most countries, with a 5‐year overall survival of 18.4%.^1^, ^2^, ^3^ Unfortunately, ∼75%–80% of LC patients are first diagnosed with advanced or distant stages due to ambiguous clinical symptoms and inadequate screening methods.^4^, ^5^ The NSCLC is closely associated with different risk factors, including genetic mutations, tobacco consumption, chronic or dysregulated inflammation, and immune dysfunction that may increase the incidence of lung cancer.^2^, ^6^ Although surgical and medical treatments of NSCLC have advanced, the response rates of the current therapeutic strategies are considered abysmal, even leading to hyperprogressive disease.^7^, ^8^ Hence, it has strategic significance to improve the understanding of pathogenesis and identify new molecular signatures that promote lung cancer progression for early diagnosis and better treatments of NSCLC.

Nowadays, the gut microbiome is recognized as the ‘second genome’ of humans.^9^, ^10^ It has been found that the diversity of the gut microbiota is closely related to cancer initiation, progression, and response to therapy.^11^ Recently published studies in animal models and humans have confirmed that the gut microbiome alters the tumour microenvironment and the circulating metabolites that in turn affect the general host physiology. For example, the imbalance of gut microbiota contributes to the metastasis‐related secretory protein cathepsin K secretion by mediating toll‐like receptor 4 (TLR4)‐dependent M2 macrophage polarization of tumour‐associated macrophages.^12^ In addition, gut microbiota alteration has resulted in an increased growth of intracranial glioma.^13^ Until now, there has only been a limited study of the gut microbiome in NSCLC. Zheng et al. confirmed that early‐stage LC patients have a significant alteration in the structural composition of microbiota compared with the controls, and the specific microbiota spectrum has been proved to be of importance in the prediction of early‐stage LC incidence.^14^ However, the disturbances of the gut microbiome reported in these studies mainly focus on the microbiota signature at a single‐omics level; thus it is difficult to achieve a comprehensive elucidation of the pathophysiological procedure of NSCLC. Further insight into the systematic biological changes of the microbiome, metabolome, and proteome for NSCLC may help in understanding the development and progression of NSCLC.

As we have known, systems biology focuses on the integration of the multiple physical changes in the organism, including genes, proteins, and metabolites at different molecular levels.^15^ The ongoing omics‐based analyses, including genomics, transcriptomics, proteomics, and metabolomics, have been widely used in various diseases^16^ such as cancer,^17^ diabetic nephropathy,^18^ and cardiovascular disease.^15^ Among them, proteomics and metabolomics are the most frequently used ‘omic’ techniques. Proteomics is used to identify significantly changed proteins that represent the contents of cells, tissues, organisms, or biofluids.^16^, ^19^ At present, liquid chromatography–mass spectrometry (LC–MS) is a widely used method of proteomics studies for exploring multiple diagnostic markers in cancers. Integrative proteomics on lung adenocarcinoma (LUAD) has revealed the plasma protein level of heat shock protein‐90β as a potential prognostic biomarker.^20^ Metabolomics is focused on the concentration of endogenous metabolites in bio‐fluids and/or tissues.^16^, ^21^ In contrast to proteomics, metabolomics studies can help to directly point out the unpleasant change in the human body organism, and differential metabolites are widely accepted as non‐invasive and sensitive markers of physiological activity.^22^, ^23^ A study by Ruiying et al. has suggested that hypoxanthine, indoleacrylic acid, acylcarnitine C10:1, and lysoPC (18:2) are potential biomarkers for NSCLC.^24^ However, due to the complexity of biological samples, the metabolites appear to be readily influenced by carrying great variability and various elements, such as age, race, gender, and nutrition.^25^ Consequently, using a single omics analysis is difficult to reveal the comprehensive changes in the biological system. Thus, a holistic and integrated analysis of genome, transcriptome, microbiome, proteome, and metabolome from different layers may contribute to augmenting understanding of the predisposing causes of disease and may promote biomarker discovery.

For this reason, a combination of 16S ribosomal RNA (rRNA) sequencing analysis of the gut microbiome in faecal samples, LC/MS‐based proteomic analysis, and ultra‐performance liquid chromatography to quadrupole time‐of‐flight mass spectrometry–based metabolomics analysis was adopted to identify promising biomarkers in serum samples of NSCLC patients. Furthermore, faecal microbiota transplantation (FMT) was recruited to further confirm the research conclusion of the multi‐omics analysis. We also illuminated the central metabolites and proteins related to NSCLC, and FMT identified a critical relationship of intestinal flora in NSCLC growth through the nervonic acid/all‐trans‐retinoic acid–interleukin‐8 (IL‐8) axis. These results may help to characterize the mechanism of NSCLC pathogenesis and to identify potential biomarkers and therapeutic targets that interrupt NSCLC development.

## 2 METHODS

### 2.1 Study subjects and specimen collection

We consecutively recruited 15 healthy controls (CON) and 55 patients who were diagnosed with NSCLC made after a biopsy at Zhejiang Cancer Hospital (Zhejiang, China) from March 2019 to September 2019. The age, gender, history of smoking, tumour stage, lymph node involvement, tumour size, and tumour metastasis were recorded. The clinicopathological information of the 55 early NSCLC patients included in the study is illustrated in Table S1. All patients with other coexisting malignant tumours and healthy controls using antibiotics within 2 months were excluded. All the healthy volunteers had normal bowel habits. For all subjects, after an overnight fast (≥8 h), blood samples from all volunteers were collected on the day after admission. Meanwhile, fresh stool specimens were self‐collected after defecation at the hospital using a special faecal collection device and were transported immediately to the laboratory, followed by placement into a −80°C chamber for further omics testing.

In addition, the second cohort of 40 LC patients was recruited at the Zhejiang Cancer Hospital (Zhejiang, China). All LC patients and healthy control volunteers voluntarily joined this study with informed consents. Then, 40 pairs of LUAD tissues (T group) and adjacent non‐cancerous tissues (N group) were collected from the participants for further metabolomics analysis. These studies were ratified by the Ethics Committee of the Zhejiang Cancer Hospital (No. IRB‐2018‐219).

### 2.2 16S rRNA gene‐sequencing

DNA of faecal microorganisms was separated and then sent to the G‐BIO Biotechnology Co., Ltd. (Hangzhou, China) for sequencing. Amplified reactions were performed using a polymerase chain reaction (PCR) instrument (Light Cycler 96, Roche, Basel, Switzerland) and quantified using QuantiFluor‐ST (Promega, USA). Then, the constructed library was analysed by the Illumina MiSeq platform (Illumina, CA, USA).^26^ Operational taxonomic units (OTUs) with ≥97% similarity were determined using VSEARCH software (v2.0.3) and annotated using the Ribosomal Database Project classifier.^27^ At last, the functions of intestinal flora were conjectured by using PICRUSt software.^28^

### 2.3 Serum‐ and tissue‐based metabolomics analysis preparation

A volume of 100‐μl serum from each sample was added to 100 μl of 2‐chlorobenzylamine in methanol solution. The mixture was vortexed, incubated, and centrifuged. The supernatant was filtered, dried, and resuspended for subsequent LC–MS analysis.

For tissue‐based metabolomics analysis, 10‐mg LC tissue samples were placed in 400‐μl precooled methanol, homogenized and centrifuged at 4°C for 15 min at 12 000 rpm. Subsequently, 200 μl of the supernatant was collected and 200 μl of water was added to the mix. Next, the mix was freeze‐dried, resuspended, and then transferred for LC–MS analysis.

### 2.4 Metabolomics and data analysis

Metabolomics analysis of processed serum and tumour samples was performed on Thermo Vanquish system. The electrospray ion trap tandem mass spectrometry experiments were executed on a Thermo Scientific Q Exactive Focus mass spectrometer. The LC–MS raw data of serum and tumour samples were converted to an mzXML format by the ProteoWizard software (v3.0.8789, Palo Alto, CA, USA). Data pretreatment was performed using package XCMS in R‐3.3.2. The potential metabolite was appraised by using the MassBank database (http://www.massbank.jp/), LipidMaps database (http://www.lipidmaps.org), mzCloud database (https://www.mzcloud.org), and the in‐house library of Suzhou SmartNuclide. Co. Ltd. (Suzhou, China). Differential metabolites among groups were screened out via VIP (variable importance value) >1 and a value of *p* < .05 based on a Student's *t*‐test and plotted by the Pheatmap package in R (v3.3.2). MetaboAnalyst 4.0 (http://www.MetaboAnalyst.ca/) was used to analyse related metabolic pathways of the identified differential metabolites. Additionally, receiver‐operating characteristic (ROC) curve analysis was performed to assess the area under curve (AUC), sensitivity, and specificity of individual metabolites within the tissue‐based metabolomics data set.

### 2.5 LC–MS/MS‐based proteomics

All serum samples were prepared by the Pierce TOP 12 Abundant Protein Depletion Spin Columns (Thermo Scientific) to remove the 12 most abundant proteins in the serum according to the user manual. Then proteins were denatured, reduced, and digested, and peptides purified on a homemade reverse‐phase C18 column in a pipet tip. The MS analysis was performed in a DDA with full scans (*m*/*z* 350–1500) acquired using an Orbitrap mass analyser.

### 2.6 Identification of DEPs and bioinformatic analysis

Proteome Discoverer (v.2.4, Thermo, America) was employed to search all of the raw data thoroughly against the UniProt database. The search parameters were set as follows: type of quantification of precursor quantification, max missed cleavage sites of 2, peptide mass tolerance of ±20 ppm, a fragment mass tolerance of .05 Da, dynamic modification of oxidation/+15.995 Da (M), N‐terminal modification of acetyl/+42.011 Da (N Terminal), static modification of carbamidomethyl/+57.021 Da (C), and a peptide false discovery rate of ≤.01. *p* < .05 and fold‐changes lower than or higher than 1.5 were considered to be differentially expressed proteins (DEPs). Volcano plot and heat map of the DEPs were visualized using the ‘limma’ package of R. To further analyse the biological meaning of DEPs, the clusterProfiler package of R was used to carry out GO and KEGG analysis. A protein–protein interaction (PPI) network was built by using the STRING database^29^ to observe the interactions between DEPs.

### 2.7 Gut bacteria, metabolomics, and proteomics data integration

To further analyse the correlations between intestinal microorganisms and serum metabolites, as well as the correlations between selected DEPs and serum metabolites, a Spearman's correlation analysis was performed by using psych package in R, and the visual presentation of their correlations was shown by a heat map using the ggplot2 package in R.

### 2.8 Experimental mice and faecal microbiota transplantation

Male C57BL/6 mice (8‐week old) were obtained from Shanghai SLAC Laboratory Animal Co. Ltd. and were housed in an SPF environment with a 12‐h light/12‐h dark cycle and fed free with standard diet and water. Experiments were approved by the Animal Care and Use Committee of Zhejiang Chinese Medical University (Zhejiang, China). The mice were treated with an antibiotic cocktail at a dose of 10 ml/kg twice per day for 14 days in drinking water to deplete the commensal gut microbiota before human stool transplantation experiments. Fresh stools were collected from volunteers and then homogenized at a concentration of 20 mg/ml. A total of 200 μl of the faecal supernatant was given to microbiota‐depleted mice for 21 days/twice per day through oral gavage. The *P. copri* group was given oral gavage with *P. copri* (Bio‐70151, Biobw Biotechnology Co., Ltd., Beijing, China) at a dose of 2 × 10^8^ colony‐forming units per 200 μl suspended in sterile anaerobic PBS twice per day, and the same volume of heat‐killed *P. copri* as the control for 3 weeks. Tumour‐bearing mice in the Lewis group were established by subcutaneous injection of 2 × 10^5^ Lewis lung cancer (LLC) cells suspended in 200‐μl growth medium into the right flank of microbiota‐depleted mice. Another tumour‐bearing mice cohort was randomized into four groups: (1) tumour control, (2) *P. copri*, (3) combination of *P. copri* and 150 mg/kg/day nervonic acid (B28317, Yuanye Co., Ltd., Shanghai, China), and (4) a combination of *P. copri* and 10 mg/kg/day trans‐retinoic acid (ENZO‐BML‐GR100‐5000, Shanghai ZZBIO CO., Ltd., Shanghai, China). The tumour volume was measured weekly by a digital caliper and was calculated with the formula *V*  =  (length × width^2^)/2. On Day 22, the blood samples were collected from retro‐orbital venous and centrifuged at 3000 rpm/min for 15 min at 4°C. All mice were sacrificed humanely, and the tumours were removed and immediately weighed. Lung and tumour samples were obtained for histological assessment. To assess intestinal inflammation, the intestine and lung tissues were also collected for further quantitative real‐time PCR and Western blot analysis, respectively.

### 2.9 ELISA and flow cytometry

The serum samples were used to measure levels of inflammatory cytokines, including TNF‐α, lipopolysaccharide binding protein (LBP), CD14 molecule (CD14), IL‐8, and C‐reaction protein (CRP) by using Quantikine enzyme‐linked immunosorbent assay (ELISA) kits (Mei Mian Biotechnology Co., Ltd., Jiangsu China). Flow cytometry was used to measure the T lymphocyte subtypes (CD3+, CD4+, and CD8+) in the peripheral blood of the mice.

### 2.10 Histological analysis

Lung and tumour tissue samples from the mice were cut into 4‐μm thick sections. Then, the sections were stained with haematoxylin–eosin (H&E). Finally, the sections were mounted with neutral gum and captured by an Olympus microscope (Olympus Corp., Tokyo, Japan).

### 2.11 Quantitative real‐time PCR assay

Total RNA of intestine tissues (100 mg) was acquired using TRIzol (Invitrogen, USA). Quantitative real‐time PCR was then performed with Hieff qPCR SYBR Green Master Mix (YEASEN, Shanghai China) on an ABI 7500 Fast Real‐Time PCR system. The PCR primers are as follows: IFNG (forward 5′‐TCGGTAACTGACTTGAATGTCCA‐3′ and reverse 5′‐TCGCTTCCCTGTTTTAGCTGC‐3′), IL‐18 (forward 5′‐GTGAACCCCAGACCAGACTG‐3′ and reverse 5′‐CCTGGAACACGTTTCTGAAAGA‐3′), IL‐1β (forward 5′‐GAAATGCCACCTTTTGACAGTG‐3′ and reverse 5′‐TGGATGCTCTCATCAGGACAG‐3′), IL‐8 (forward 5′‐ACACTGCGCCAACACAGAAATTA‐3′ and reverse 5′‐TTTGCTTGAAGTTTCACTGGCATC‐3′), TNF‐α (forward 5′‐AGCCGATGGGTTGTACCT‐3′ and reverse 5′‐TGAGTTGGTCCCCCTTCT‐3′), TLR4 (forward 5′‐AAGTTATTGTGGTGGTGTCTAG‐3′ and reverse 5′‐GAGGTAGGTGTTTCTGCTAAG‐3′), MyD88 (forward 5′‐CTCCATTCCTCCTCCAGACACT‐3′ and reverse 5′‐AAGGAGAGGCAGTTTGGCTTC‐3′), NF‐κB (forward 5′‐TTACGGGAGATGTGAAGAT‐3′ and reverse 5′‐ATGATGGCTAAGTGTAGGA‐3′), and GAPDH (forward 5′‐GGAGCGAGATCCCTCCAAAAT‐3′ and reverse 5′‐GGCTGTTGTCATACTTCTCATGG‐3′). The results were normalized against GAPDH and were calculated with the 2^−ΔΔ^ *^CT^* method.

### 2.12 Western blot analysis

Lung samples were homogenized and qualified with a BCA detecting kit (Beyotime, China). Protein lysates of 30 μg were separated by SDS‐PAGE and transferred onto the PVDF membrane (Millipore, USA). After being blocked with 5% (w/v) nonfat milk, the membranes were then incubated with primary antibodies against TLR4 (1:500, ab13556, Abcam), MyD88 (1:1000, ab219413, Abcam), NF‐κB p65 (1:2000, ab32536, Abcam), Bax (1:1000, ab182734, Abcam), Bcl‐2 (1:2000, ab182858, Abcam), and β‐actin (1:5000, D191047, Sangon Biotech, China) at 4°C overnight. Next, the membranes were incubated with HRP‐conjugated goat anti‐rabbit secondary antibody (1:2000, ab7090, Abcam) for 1 h at room temperature. Finally, the optical density of bands was measured via ImageJ software.

### 2.13 Statistical analysis

All data are shown as mean ± standard deviation. Significant differences between groups were analysed by two‐tailed Student's *t*‐test or one‐way analysis of variance followed by Bonferroni post hoc tests. A value of *p * < .05 was considered significant.

## 3 RESULTS

### 3.1 Analysis of gut microbiota

**FIGURE 1:**
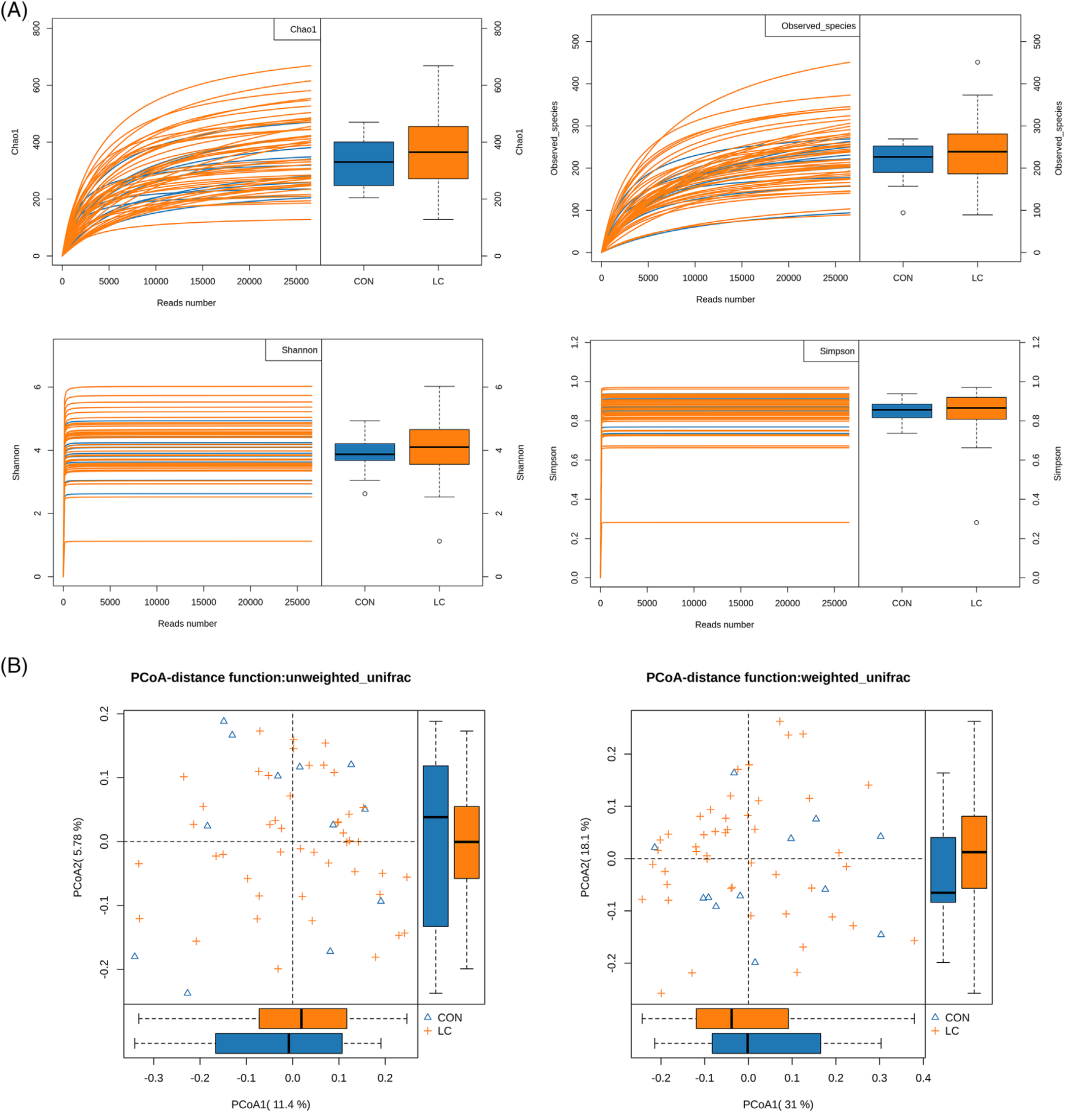
The preliminary comparison on diversity of gut microbiota in human adults withNSCLC and healthy controls. (A) Rarefaction curves for the reads number in the NSCLC (*n* = 46) and control (*n* = 12) groups after 100 random samplings. Each curve represents a sample, marked with blue and yellow colours, respectively. Indexes of Chao 1, Observed_species, Shannon, and Simpson were used to assess the α‐diversity of the gut microbiota between LC patients and healthy controls. (B) Principal coordinates analyses based on weighted (left) and unweighted (right) UniFrac distances between gut bacterial communities of LC and healthy control patients

The intestinal flora composition of NSCLC patients was investigated by measuring faecal 16S DNA. A total of 2112 130 reads and 2104 029 (99.6%) effective reads were obtained from 58 samples (12 healthy controls and 46 NSCLC patients) from two groups, with an average of 36 276 reads per sample (Table S2). As illustrated in Figure 1A, the rarefaction curves revealed that the curve of each sample gradually flattened out with the increasing number of reads, suggesting that the sequencing data were sufficient to perform further analysis. Moreover, the α‐diversity indexes, including Chao 1, Observed_species, Shannon, and Simpson were used to assess the diversity and community richness in the samples. Unexpectedly, there were no significant differences in α‐diversity indexes between the LC and healthy control groups in this study. Additionally, β‐diversity analysis was used to reveal the gut microbiota structure of LC patients. The PCoA was analysed by the weighted and unweighted UniFrac metric. As shown in Figure 1B, the results demonstrated the gut microbiota structure between the LC group and the control group with no differences.

In total, 3275 OTUs (Table S3) were annotated for subsequent analyses, including 16 phyla (Table S4), 31 class (Table S5), 55 order (Table S6), 118 family (Table S7), and 289 genera (Table S8) of gut microbes. A total of 13 and 199 OTUs were shared by the two groups at the phylum and genus level, respectively. As shown in Figure S1A, the Venn diagrams also display the difference between each group, exhibiting specific 69 OTUs in NSCLC and 21 OTUs in the control group, respectively. The results of the analysis at the phylum level are illustrated in Figure S1B. Four phyla, including *Actinobacteria*, *Proteobacteria*, *Firmicutes*, and *Bacteroidetes*, were measured in all of the faecal samples. Among them, in the NSCLC group, the abundance of *Actinobacteria* and *Proteobacteria* was decreased and that of *Firmicutes* and *Bacteroidetes* was increased. In addition, the most abundant genera in the LC group were *Megamonas*, *Gemmiger*, *Roseburia*, *Prevotella*, and *Bacteroides*. Furthermore, the results of the correlation analysis of several taxa in LC patients are shown in Figure S1C. Thus, we found that *Prevotella* and *Collinsella* had the strongest negative correlation; *Gemmiger* and *Roseburia* had the strongest positive correlation in this study.

**FIGURE 2:**
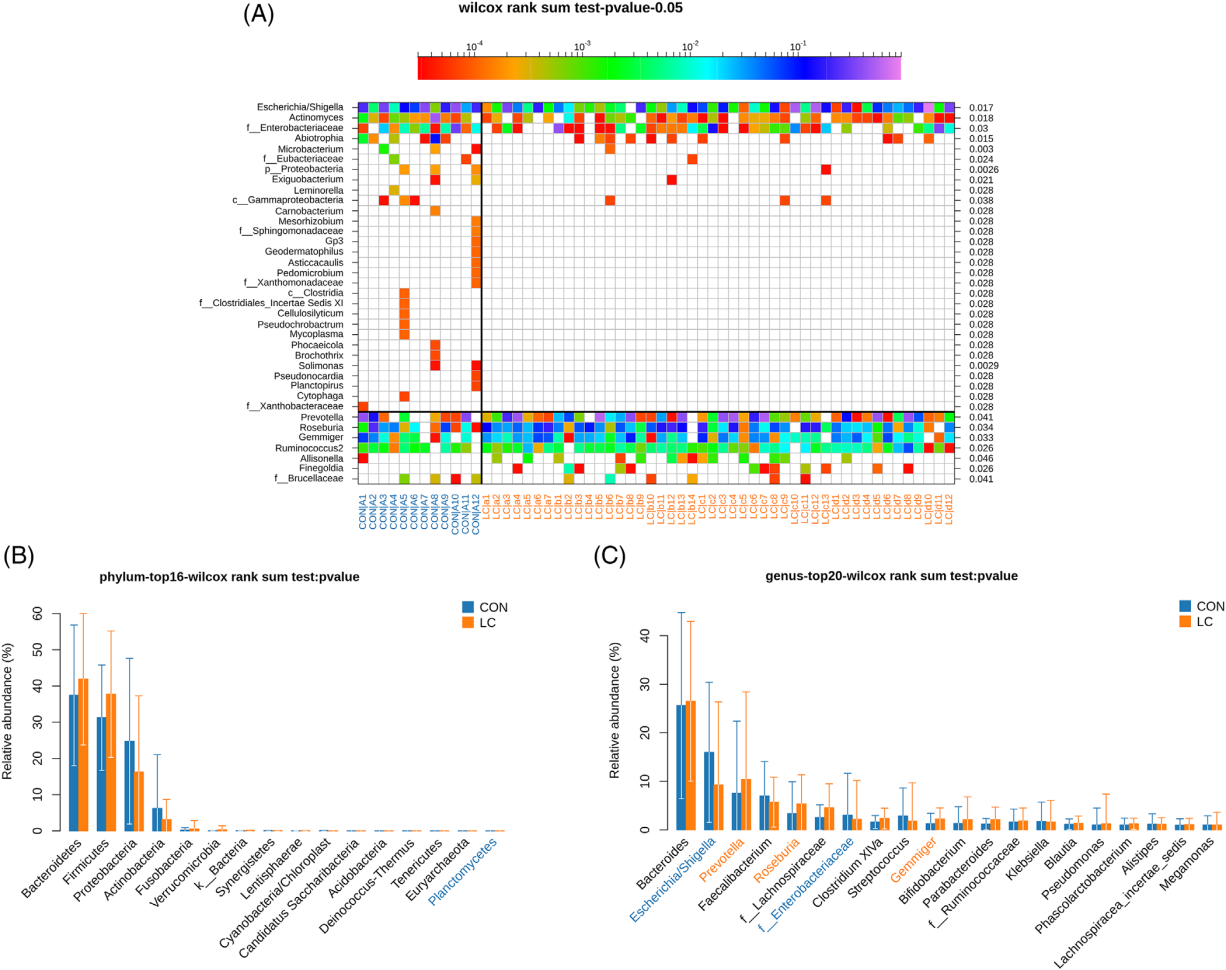
Compositional differences of the gut microbiota between NSCLC and healthy control groups. (A) Heat map of gut microbiota showed that the genus in LC patients is different from healthy controls (Wilcoxon rank‐sum test). Comparison of relative abundance of significantly altered bacterial taxa, including phylum (B) and genus (C) levels between LC patients and healthy controls. Wilcoxon rank‐sum test was employed to measure the significance between groups.

From the community heat map diagram and the box plots, we soon realized that the chief genera in NSCLC patients were *Planctomycetes*, *Escherichia/Shigella*, *Prevotella*, *Roseburia*, f_Enterobacteriaceae, and *Gemmiger* (Figure 2A–C). Among them, the abundance of *Prevotella*, *Roseburia*, and *Gemmiger* was markedly increased in NSCLC patients. Between LC patients and healthy controls, seven bacteria were discriminative in healthy controls from the family level to the genus level, as depicted in the cladogram (Figure S2A). Compositional analysis by LefSe revealed that faecal samples of healthy controls were enriched with the genus *Gardnerella*, *Exiguobacterium*, *Microbacterium*, and *Solimonas*, whereas there was no predominant microbiota in the NSCLC group (Figure S2B). The PICRUSt method was selected to predict the KEGG pathways between the microbiome of NSCLC patients and healthy controls. In the NSCLC group, it is noteworthy that the microbiome takes part in sporulation and thiamine metabolism (Figure S2C). Taken together, these results demonstrate that the genera *Prevotella*, *Roseburia*, and *Gemmiger* could be associated with the pathological process of NSCLC.

### 3.2 Significant differences in the serum‐based metabolomics of NSCLC patients

**FIGURE 3:**
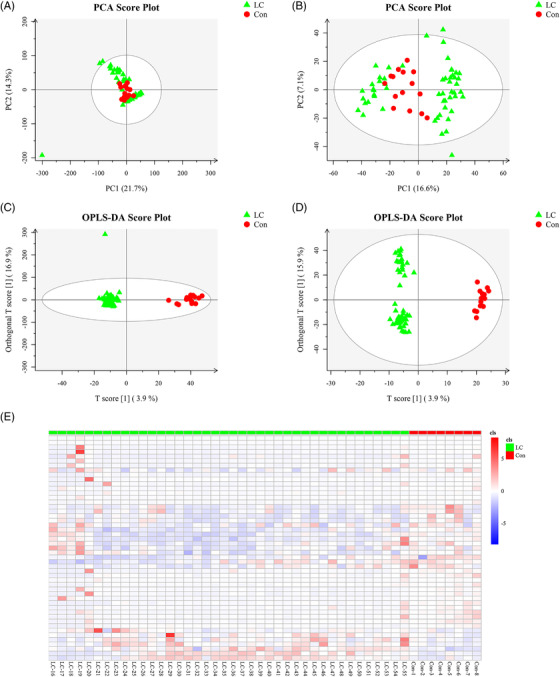
The metabolic profiles of serum samples in LC patients. PCA score plot of serum metabolite profiling between the LC and control groups in (A) positive mode (seven principal components, *R*2 × .525) and (B) negative mode (10 principal components, *R*2 × .509). OPLS‐DA score plot of serum samples and the differentiation of the metabolome between two groups in (C) positive mode (four principal components, *R*2 × .43, R2Y .974, *Q*2 .745) and (D) negative mode (four principal components, *R*2 × .271, R2Y .985, *Q*2 .734). (E) The heat map shows the expressive pattern of 49 differential metabolites (VIP > 1, *p* < .05).

More than 80% of RSDs < 30% for QC samples are shown in positive (Figure S3A) and negative modes (Figure S3B), respectively. Base peak chromatograms of serum‐based metabolomics of LC patients are presented in Figure S4. A total of 13 402 precursor molecules in the positive mode and 3578 precursor molecules in the negative mode were obtained for subsequent analysis. The PCA score plot clearly showed a complete separation of the serum between the LC and control groups in positive (Figure 3A) and negative (Figure 3B) ion mode. Also, in the Orthogonal projections to latent structure‐discriminant analysis (OPLS‐DA) of LC and control groups, the score plots exhibited distinct clusters between two groups in both positive (Figure 3C) and negative modes (Figure 3D), which revealed that the metabolic patterns were remoulding in LC patients.

A total of 49 significantly changed metabolites between the LC group and control group samples were identified, as illuminated in the heat map (Figure 3E), of which, 42 showed a downward trend, and 7 showed an increasing trend (Table S9). Briefly, 49 metabolites could mainly be classified into 7 amino acids, 3 steroids, 8 fatty acids, 1 retinoid, 2 nucleosides, and 28 other classified metabolites. *m*‐Coumaric acid, 13‐l‐hydroperoxylinoleic acid, allocholic acid, 13S‐hydroxyoctadecadienoic acid, palmitic acid, 5‐hydroxyindoleacetic acid, eicosadienoic acid, pyroglutamic acid, 3‐(2‐hydroxyphenyl)propanoic acid, nervonic acid, arachidic acid, l‐glutamic acid, and oxoadipic acid exhibited lower concentrations in LC patients than in controls (Figure S5). Notably, the metabolites in serum samples were basically related to alanine, aspartate, and glutamate metabolism, arginine and proline metabolism, retinol metabolism, caffeine metabolites, d‐glutamine and d‐glutamate metabolism, and glutathione metabolism (Figure S6).

### 3.3 Metabolomics analysis of tumour samples from LC patients

**FIGURE 4:**
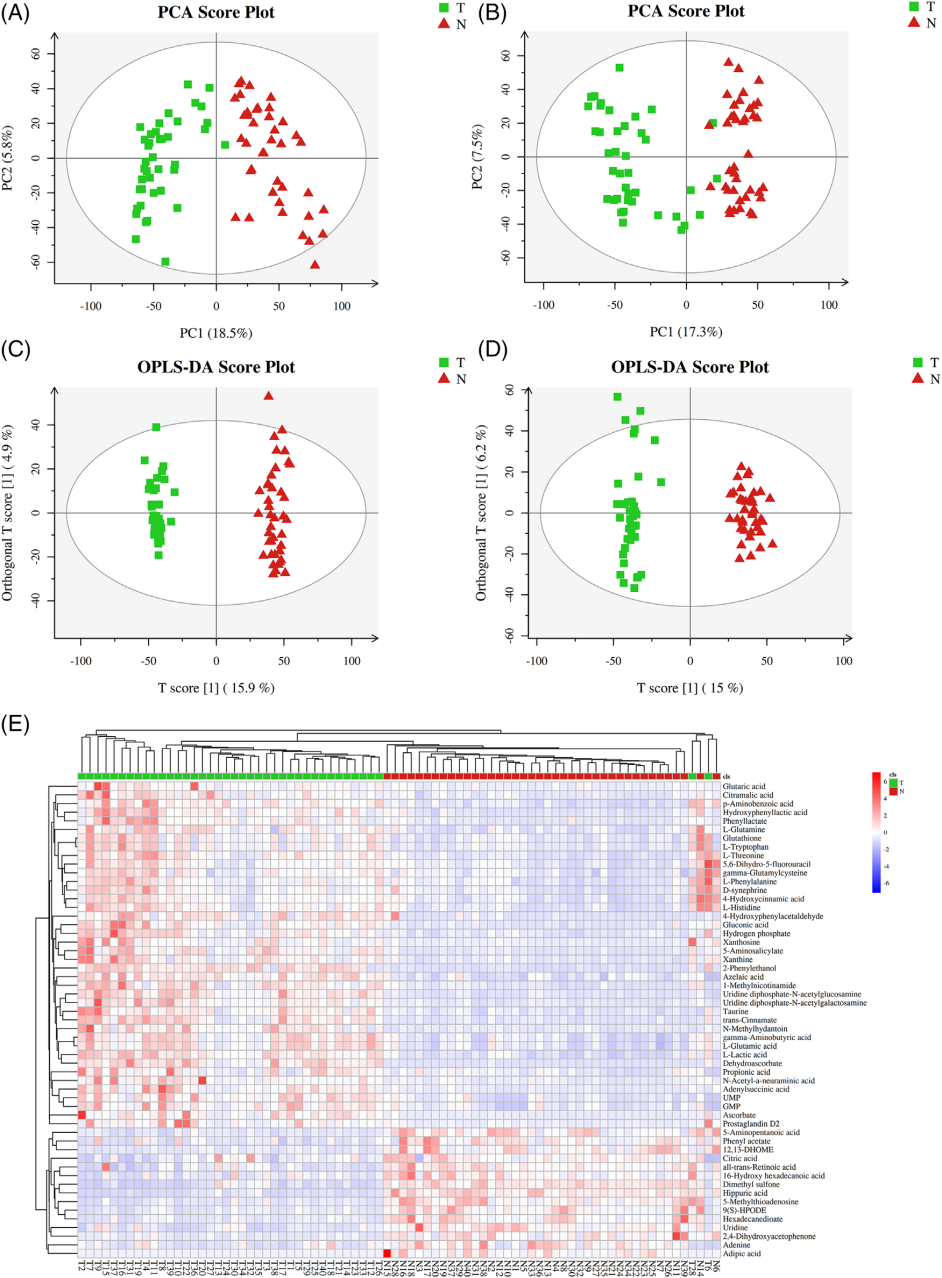
The metabolic profiles of tumour samples in LC patients. PCA score plot of tumour tissue‐based metabolite profiling between the LC and control groups in (A) positive mode (10 principal components, *R*2 × .484) and (B) negative mode (10 principal components, *R*2 × .488). OPLS‐DA score plot of tumour samples discrimination of the metabolome between two groups in (C) positive mode (three principal components, *R*2 × .255, R2Y .988, *Q*2 .901) and (D) negative mode (three principal components, *R*2 × .268, R2Y .975, *Q*2 .888). (E) The heat map shows the expressive pattern of 55 differential metabolites (VIP > 1, *p* < .05).

More than 90% of RSDs were less than 30%, suggesting that the acquired data were reasonable for further analysis (Figure S7). The total ion chromatograms of tissue‐based metabolomics analysis are shown in Figure S8. A total of 23 683 metabolite ions were extracted from all LC cancerous and non‐cancerous tissue samples, and after normalization, 22 291 ions were remained. The PCA (Figure 4A,B) and OPLS‐DA (Figure 4C,D) results in positive and negative ion modes showed an obvious separation of the tissues between the non‐cancerous group and the tumour groups.

A total of 55 metabolites were identified, among which 40 metabolites were significantly increased, and 15 metabolites showed a downward trend in cancerous tissues of LC patients (Table S10). The heat map revealed that LC cancerous tissue has a different metabolic pattern from non‐cancerous tissue based on these 55 differential metabolites (Figure 4E). In particular, the levels of *p*‐aminobenzoic acid, azelaic acid, 4‐hydroxycinnamic acid, l‐lactic acid, *N*‐acetyl‐α‐neuraminic acid, gluconic acid, hydroxyphenyllactic acid, gamma‐aminobutyric acid, adenylsuccinic acid, citramalic acid, glutaric acid, and propionic acid were significantly increased in LC patients, whereas hippuric acid, adipic acid, 5‐aminopentanoic acid, 16‐hydroxy hexadecanoic acid, and citric acid were evidently reduced, as they could play a decisive role in the development of LC (Figure S9).

As shown in Figure S10, MetaboAnalyst 4.0 indicated that 55 endogenous metabolites‐related metabolic pathways were GABAergic synapse, intestinal immune network for IgA production, glutamatergic synapse, small cell lung cancer, glutathione metabolism, nicotine addiction, African trypanosomiasis, gastric cancer, Th17 cell differentiation, alanine, aspartate and glutamate metabolism, d‐glutamine and d‐glutamate metabolism, and ferroptosis. To further elucidate the common characteristics, the overlapping metabolites and metabolic pathways were also identified using a Venn diagram. A total of 28 overlapping metabolic pathways were screened, as shown in Table S11. We screened six common metabolites in serum‐ (Figure S11A) and tissue‐based metabolomics (Figure S11B), respectively, including 9(S)‐HPODE, l‐glutamic acid, xanthine, l‐glutamine, all‐trans‐retinoic acid, and gamma‐glutamylcysteine. Interestingly, results showed that 9(S)‐HPODE and all‐trans‐retinoic acid showed a similar trend of downregulation, and xanthine showed a similar trend of upregulation in LC patients in both metabolomic analyses.

**FIGURE 5:**
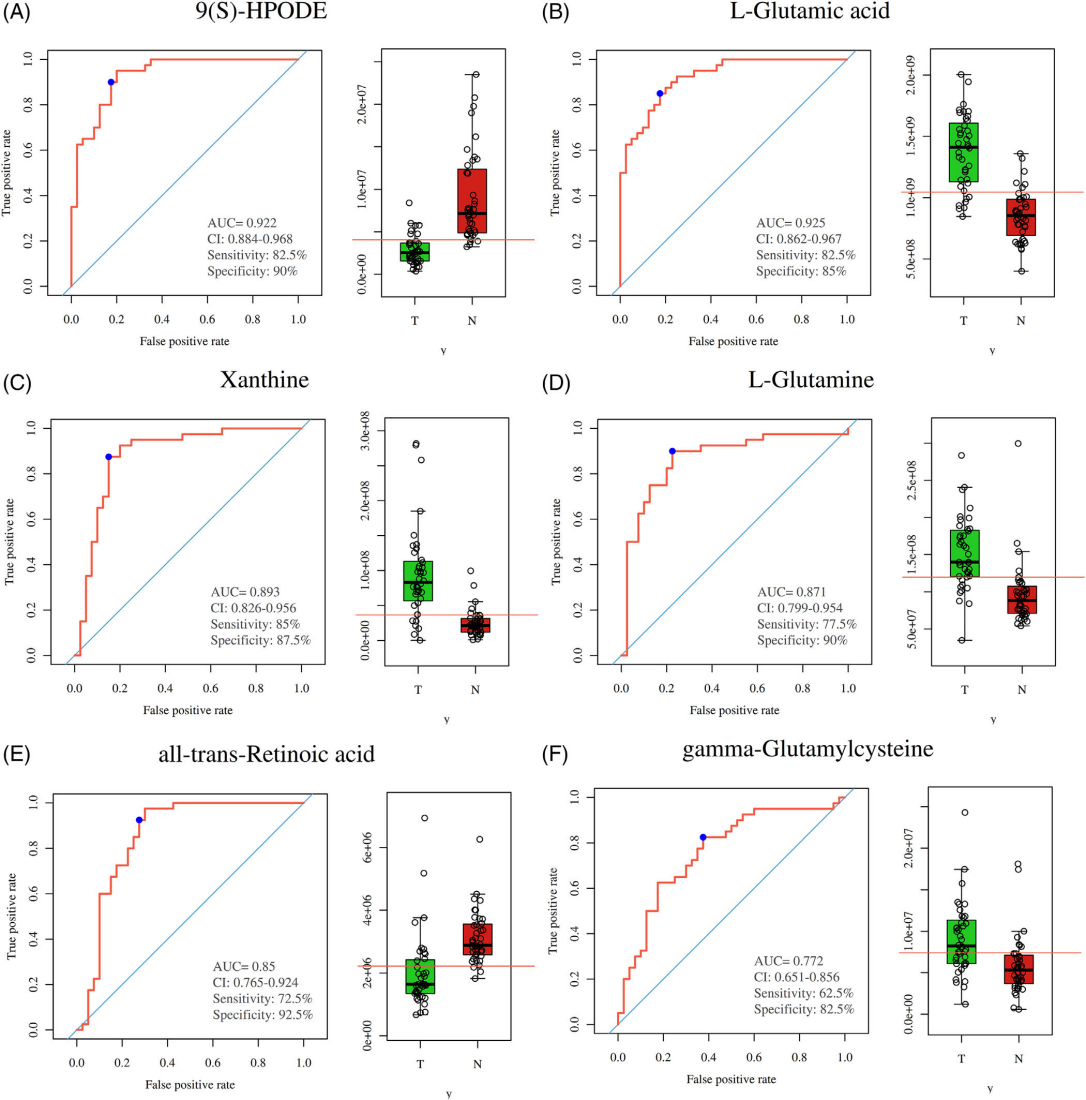
Overlapping metabolites in both serum‐ and tissue‐based metabolomics. Receiver operating characteristic curves of (A) 9(S)‐HPODE, (B) l‐glutamic acid, (C) xanthine, (D) l‐glutamine, (E) all‐trans‐retinoic acid, and (F) gamma‐glutamylcysteine in tissue‐based metabolomics. Control group versus LC group; N group versus T group, *p < .05, **p < .01, ***p < .001

To explore the role of overlapping metabolites on the predictive performance of metabolites in LC, we determined the AUC of the ROC curve on each metabolite (Table S12). Among them, the predictive performance of six overlapping metabolites was selected as diagnostic biomarkers with satisfactory predictive accuracy, especially all‐trans‐retinoic acid (Figure 5). The previous results may illustrate that all‐trans‐retinoic acid may be involved in LC progression.

### 3.4 Protein identification and bioinformatics analysis

**FIGURE 6:**
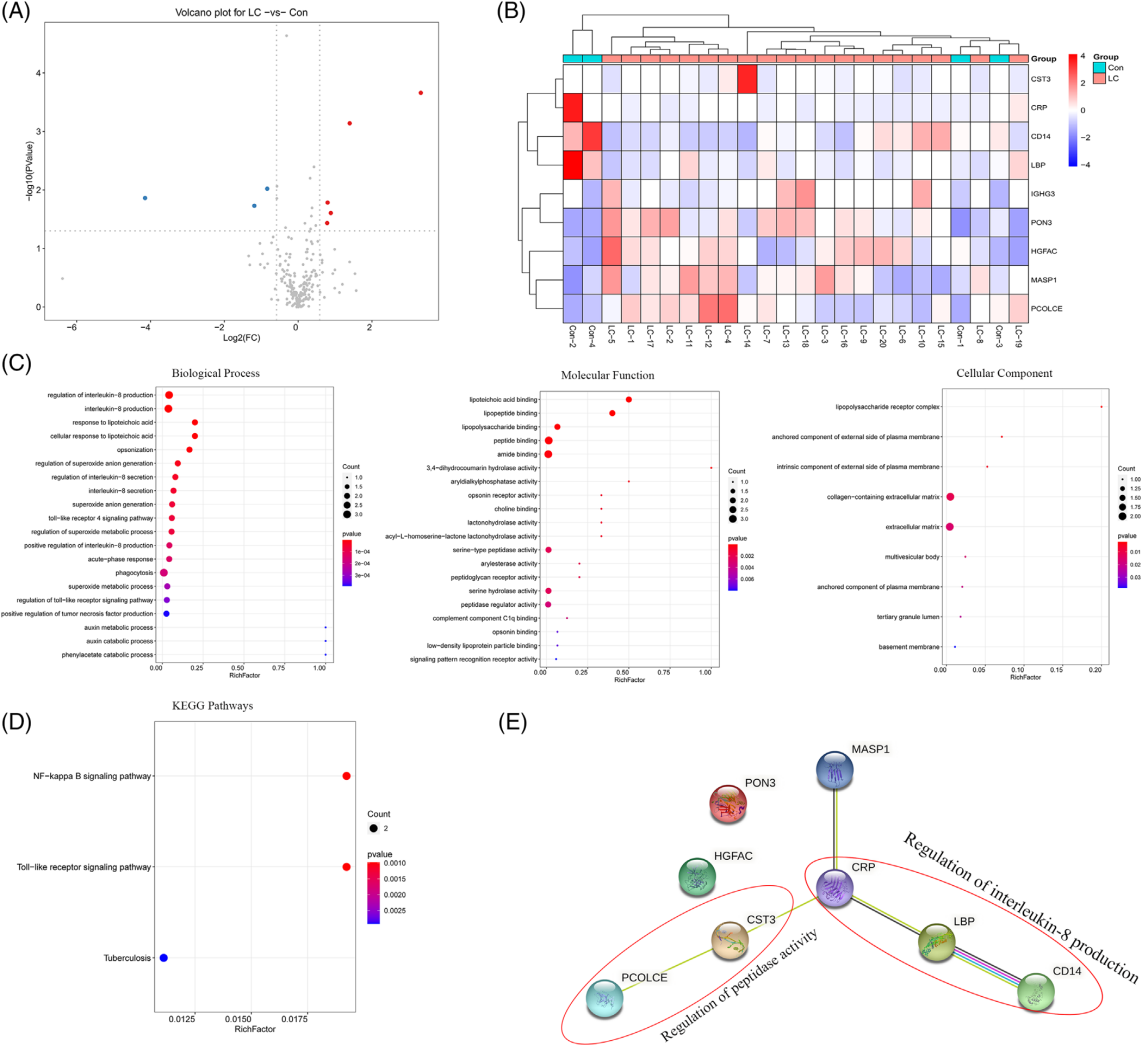
Proteomics analysis of serum samples in LC patients: (A) volcano plot of differentially expressed proteins (DEPs). The threshold set for DEPs was a fold‐changes (FC) >1.5 and a *p* < .05. Five proteins are upregulated (red) and three proteins are downregulated (green). (B) Heat map of the nine DEPs between the LC and control groups, with folds >±1.5 and *p* < .05. The columns represent DEPs and the rows indicate the samples. The colours from violet to red indicate the increasing content of DEPs. (C and D) GO analysis and KEGG pathway enrichment analysis of the nine DEPs. Terms in the same category were ranked based on the *p*‐values. (E) protein–protein interaction (PPI) networks of the identified DEPs. Red ellipses surrounded dots are associated with the regulation of interleukin (IL)‐8 production and peptidase activity, respectively.

Serum‐based proteomics analysis found that 392 proteins were identified, of which 225 proteins were identified and quantified with at least one unique peptide and had quantitative values in at least 50% of the samples (Table S13). Importantly, a total of eight DEPs were screened out between the LC and control groups, respectively (Figure 6A,B, Table S14).

To explore the biological functions of those DEPs, a bioinformatics analysis of these DEPs was performed (Figure 6C). Significantly enriched biological process (BP) included a regulation of IL‐8 production, cellular response to lipoteichoic acid, a regulation of superoxide anion generation, TLR4 signalling pathway, and a positive regulation of TNF production. DEPs were primarily concerned with lipoteichoic acid binding, lipopeptide binding, lipopolysaccharide binding, peptide binding, amide binding, serine hydrolase activity, and peptidase regulator activity. In addition, the most enriched GO terms in the cellular compartment category were the lipopolysaccharide receptor complex, anchored/intrinsic component of the external side of the plasma membrane, and collagen‐containing extracellular matrix. KEGG analysis revealed that the DEPs were only enriched in the NF‐κB and TLR pathways (Figure 6D).

The PPI network was consisting of eight nodes and five edges in the STRING database. Among the eight nodes, CRP, LBP, and CD14 were mainly involved in the regulation of IL‐8 production; cystatin C (CST3) and procollagen C proteinase enhancers (PCOLCE) were associated with the regulation of peptidase activity (Figure 6E). These findings imply that CRP, LBP, and CD14 may mediate the IL‐8 production, which aggravates tumour growth in LC.

### 3.5 Correlation among gut microbiota, serum‐based metabolomics, and proteomics

As shown in a heat map (Figure S12), nervonic acid and all‐trans‐retinoic acid were negatively correlated with *Prevotella*. Oxoadipic acid, ornithine, and l‐glutamic acid were negatively correlated with the abundance of *Gemmiger*. 3‐(2‐Hydroxyphenyl)propanoic acid was negatively correlated with *Roseburia*, but beta‐d‐fructose and tryptamine were correlated positively with the *Roseburia*. Similarly, the potential relationships between DEPs and serum metabolites were evaluated. Moreover, we found that the level of CD14 was positively correlated with leukotriene C4, palmitic acid, eicosadienoic acid, arachidic acid, 5‐hydroxyindoleacetic acid, and 4‐hydroxytamoxifen, but negatively correlated with 9,10‐12,13‐diepoxyoctadecanoate and 9(S)‐HPODE; LBP showed highly positive correlations with cortolone, whereas 9(S)‐HPODE had the opposite correlation with the DEP (Figure S13). More importantly, there were no significant correlations between DEPs and nervonic acid or all‐trans‐retinoic acid. To a certain extent, we found that nervonic acid and all‐trans‐retinoic acid were negatively associated with CD14, CRP, and LBP, thereby indicating that understanding the relationships between these DEPs, bacteria, and metabolites may broaden our knowledge of the development of LC.

### 3.6 Effects of FMT and *Prevotella copri* on inflammation and immune function in mice

**FIGURE 7:**
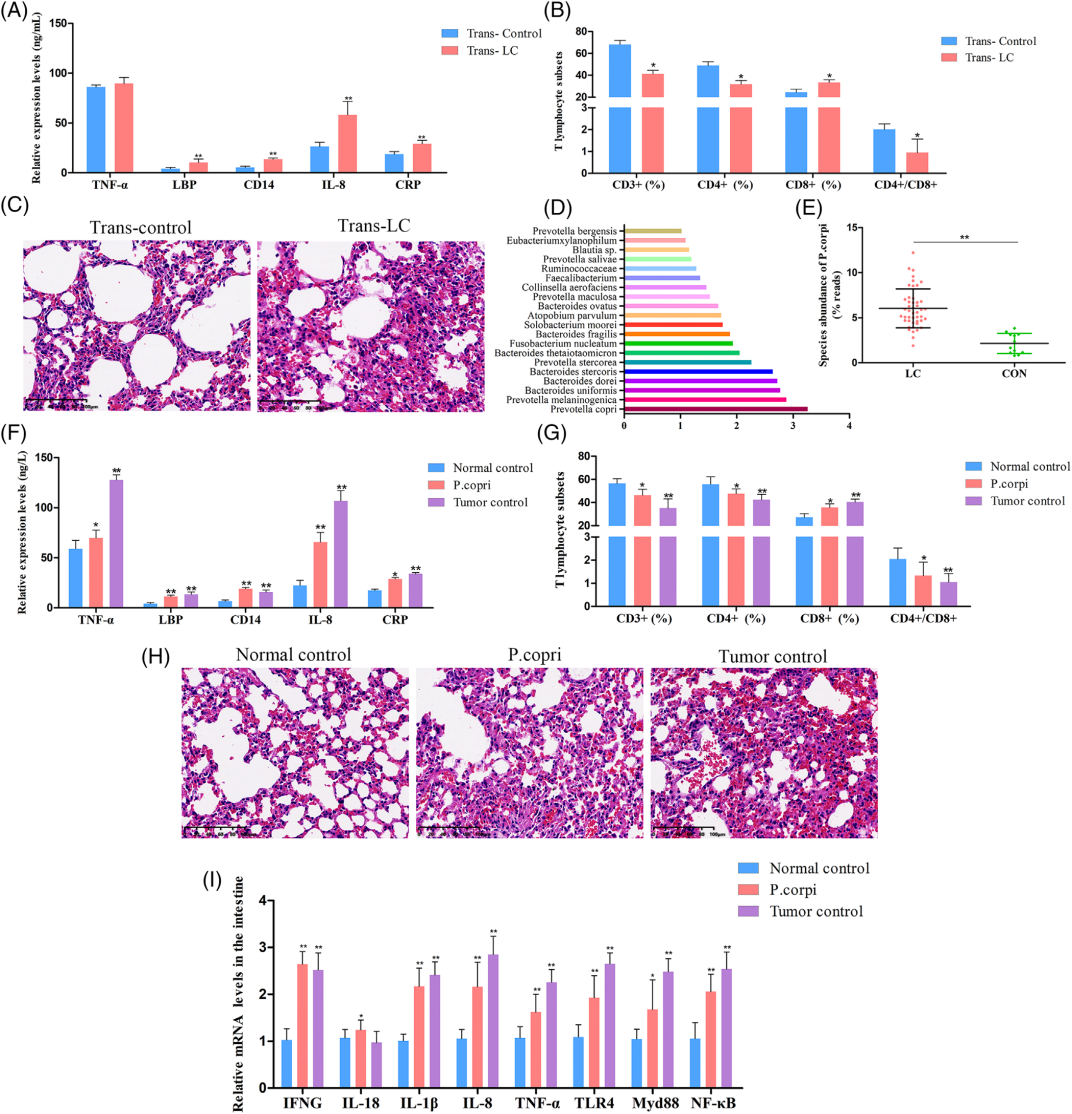
Effects of LC faecal microbiota or *Prevotella copri* transplantation deteriorated inflammatory response and immunologic derangement. The mice transplanted with stool suspensions from LC patients and healthy controls were defined as trans‐LC and trans‐control, respectively. After treatment with antibiotics in drinking water for 2 weeks, the mice were given oral gavage with stool suspensions twice per day for (A)–(C). (A) Enzyme‐linked immunosorbent assays (ELISAs) were used to measure levels of TNF‐α, lipopolysaccharide binding protein (LBP), CD14, interleukin (IL)‐8, and C‐reaction protein (CRP) in serum samples. (B) The percentages of CD3^+^ T cells, CD4^+^ T cells, and CD8^+^ T cells in peripheral blood. (C) The histological analysis of lung tissues evaluated by haematoxylin–eosin (H&E) staining. (D) VIP scores of OPLS‐DA. VIP scores were used to rank the discriminating power of different taxa between the LC and control groups. If VIP>1, the taxon was considered significant in the discrimination. (E) *P. copri* species abundance in LC and healthy controls (*p* = 1.55478E−07; the *p*‐value was determined by two‐tailed Wilcoxon rank‐sum test and data are presented as means ± standard deviations). For (F)–(I), mice were divided into three groups (normal control, *P. copri*, and tumour control). For the *P. copri* group, the mice were administered *P. copri* by oral gavage for 21 days. For the tumour control group, the right flank of mice was injected subcutaneously with Lewis lung cancer (LLC) cells to prepare a subcutaneous tumour model. Heat‐killed *P. copri* was used as a control in the normal control group. (F) TNF‐α, LBP, CD14, IL‐8, and CRP levels. (G) Quantitative analysis of CD3+ T cells, CD4+ T cells, and CD8+ T cells. (H) H&E staining of representative lung tissues. Scale bar: 100 μm. (I) Relative expression of interferon‐gamma (IFNG), IL‐18, IL‐1β, IL‐8, TNF‐α, toll‐like receptor 4 (TLR4), MyD88, and NF‐κB in the intestinal tissues determined by qRT‐polymerase chain reaction (PCR). All data are presented as the mean ± standard deviations, *n* = 6, *p < .05, **p < .01, compared with the control groups.

To observe the functions of the gut microbiota on the inflammation and immune function in the host, the microbiota‐depleted mice were treated with stools from LC patients or healthy controls. As expected, in the trans‐LC group, the amounts of TNF‐α, LBP, CD14, IL‐8, and CRP were markedly increased in the serum as compared to that in the trans‐control groups (Figure 7A). Interestingly, mice transplanted with stool from LC patients showed immunity functional disorder, as revealed by decreased CD3+ and CD4+, and increased CD8+ T‐cell counts (Figure 7B). Lung sections collected from mice transplanted with healthy control stool microbiota exhibited normal bronchial, alveolar, and vascular structures, and the proliferation of bronchial epithelial cells was not observed. However, mice transplanted with stool microbiota from LC patients showed an extensive infiltration of inflammatory cells into the alveolar space, with increased mucous secretion and epithelial cell proliferation in the lung tissues (Figure 7C). In addition, the VIP score for the gut microbiota showed that *P. copri* was beneficial to the group separation (Figure 7D). Importantly, the abundance of *P. copri* was dramatically increased in the LC group compared with those in the control group (Figure 7E). Therefore, we speculated that the changes in nervonic acid and all‐trans‐retinoic acid might connect *Prevotella* dysbiosis to malignant behaviours of LC.

To address the role of *P. copri* in the pathogenesis of LC, mice were intragastrically administered *P. copri* for 21 days, and heat‐killed *P. copri‐*treated mice were used as normal control. LLC‐bearing mice were used as tumour control to assess the effects of *P. copri* on LC pathogenesis. Similarly, oral gavage with *P. copri* caused aggravating inflammation and destroyed the immune balance and lung morphology in recipient mice (Figure 7F–H). The gut microbiota may help LC pathogenesis through impact on immune responses and inflammation.^10^ Thus, intestinal immune cell‐produced cytokines, including interferon‐gamma (IFNG), IL‐18, IL‐1β, and TNF‐α, as well as TLR4/NF‐κB signalling‐related targets, were measured in the intestinal samples. As indicated in Figure 7I, significant increases in IFNG, IL‐18, IL‐1β, IL‐8, TNF‐α, TLR4, MyD88, and NF‐κB levels were found in the *P. copri* and tumour control groups. These resells suggested that the intestinal flora from LC patients, especially *P. copri*, may contribute to the chronic inflammation and immunity functional disorder of LC patients.

### 3.7 Restoration of nervonic acid and trans‐retinoic acid reversed the effects of *Prevotella copri* in LLC‐bearing mice

**FIGURE 8:**
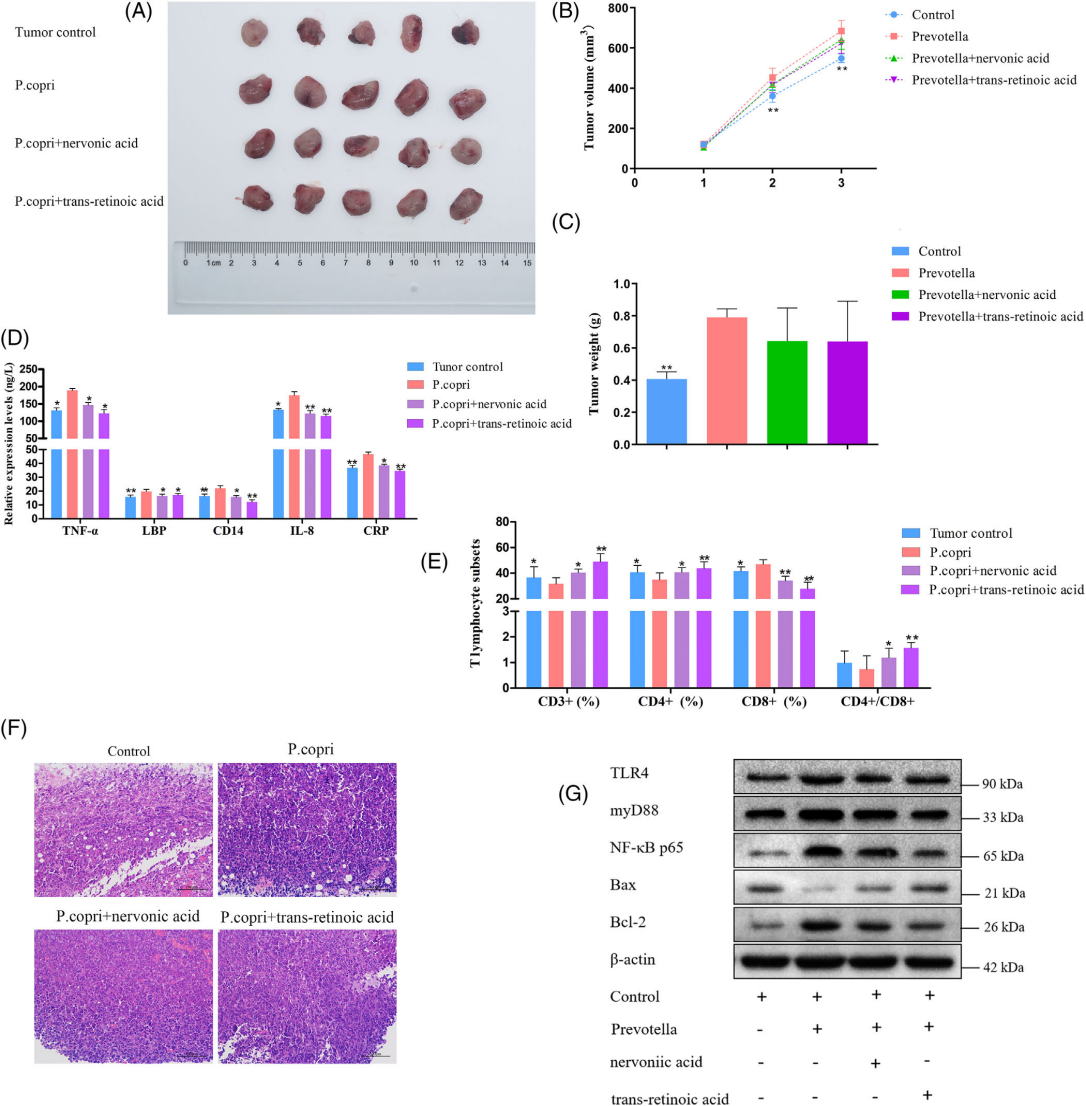
Effects of *Prevotella copri* and combination of nervonic acid or trans‐retinoic acid on Lewis lung cancer (LLC)‐bearing mice. (A) Xenograft tumours in mice from the four treatment groups (tumour control, *P. copri*, *P. copri* + nervonic acid, *P. copri* + trans‐retinoic acid) after subcutaneous injection of LLC (*n* = 5 for each group). (B and C) Volume and weight of tumours were measured in tumour control, *P. copri*, *P. copri* + nervonic acid, and *P. copri* + trans‐retinoic acid groups. (D) The expression levels of TNF‐α, lipopolysaccharide binding protein (LBP), CD14, interleukin (IL)‐8, and C‐reaction protein (CRP), as determined by enzyme‐linked immunosorbent assays (ELISAs). (E) The percentages of CD3+ T cells, CD4+ T cells, and CD8+ T cells in peripheral blood, as measured by flow cytometry. (F) Haematoxylin–eosin (H&E) staining of LLC tumours in each group (×100). (G) In lung tissues, the expression of toll‐like Receptor 4 (TLR4), MyD88, NF‐κB p65, and Bcl‐2 decreased and the expression of Bax increased after treatment with nervonic acid or trans‐retinoic acid, measured by the Western blot. β‐Actin was used as an endogenous control. The data represent the mean ± standard deviations, *p < .05, **p < .01, compared with the *P. copri* group.

Next, we investigated whether *P. copri* promotes lung cancer progression through nervonic acid and trans‐retinoic acid. *P. copri*‐treated LLC‐bearing mice were administered nervonic acid and trans‐retinoic acid for 21 days. Tumour size was measured every 7 days. Results showed that nervonic acid and trans‐retinoic acid led to a decrease in tumour size and weight; however, no obvious difference was noticed between the treated groups and *P. copri* group (Figure 8A–C). Unexpectedly, we found that the administration of nervonic acid or trans‐retinoic acid to *P. copri*‐treated LLC‐bearing mice decreased inflammatory reaction and reversed the immunologic imbalance, as revealed by a significant decrease in TNF‐α, LBP, CD14, IL‐8, and CRP levels (Figure 8D), and accompanied by marked increase in the proportions of CD3+, CD4+ T lymphocytes, and the ratio of CD4+ to CD8+ cells (Figure 8E). Unfortunately, no significant difference was found in the histopathologic examination of xenograft tumours among groups with densely packed tumour cells and apparent cell heteromorphosis (Figure 8F). To investigate the formation of inflammatory injury to the lungs, TLR4/MyD88/NF‐κB signalling pathway‐/apoptosis‐related protein levels were detected by the Western blot. The resulting data indicated that TLR4, MyD88, NF‐κB p65, and Bcl‐2 protein levels were markedly increased and Bax levels reduced after *P. copri* treatment compared with the tumour control group. Compared with the *P. copri* group, protein levels of TLR4, MyD88, NF‐κB p65, and Bcl‐2 were decreased significantly, accompanied by promoted Bax levels in the nervonic acid or trans‐retinoic acid‐treated groups (Figure 8G). Thus, nervonic acid or trans‐retinoic acid might be considered effective in the treatment of chronic inflammation and dysimmunity in LLC‐bearing mice administered with *P. copri*.

## 4 DISCUSSION

Recent evidence suggests that gut dysbiosis contributes to immune dysregulation, chronic inflammation, and progression of various tumours, including lung cancer, through the ‘gut–lung’ axis.^30^, ^31^, ^32^ In this study, we systematically investigated the gut microbiota profiles, serum‐/tissue‐based metabonomics, and serum‐based proteomics in early NSCLC patients using 16S rDNA sequencing and LC–MS approach. Our findings indicated that the gut microbiota richness and diversity had no differences between LC patients and healthy controls. This result is inconsistent with a study by Liu et al.^10^ investigating the gut microbiota in 16 healthy individuals and 30 lung cancer patients. Also, an increase in gut microbiota diversity has been found in some patients with other malignant tumours, such as thyroid carcinoma,^33^ gastric cancer,^34^ and oesophageal cancer.^35^ At the phylum level, the faecal samples of LC patients show a higher abundance in *Bacteroidetes* and *Firmicutes* compared to that of the healthy control group, similar to the study results of Yu et al.^36^ but different from that of a study by Zhuo et al.^37^ As we know, the *Firmicutes* to *Bacteroidetes* ratio is often used to determine health status and may indicate the bacterial population balance of the gastrointestinal tract.^33^ Thus, we speculated that increased *Firmicutes* to *Bacteroidetes* ratio may contribute to the pathogenesis of LC. Notably, we also found that the microbiome of LC groups demonstrates a relatively lower abundance of *Proteobacteria* and *Actinobacteria* than healthy controls. However, *Proteobacteria* is a potential pathogen, resulting in the imbalance of gut microbiota in LC patients.^10^ Similar to our study, Zhuang et al. found apparently decreased *Actinobacteria* sp. as a promising biomarker in LC.^38^ These findings may imply that the gut microbiota participated in the immune imbalances of LC.

Additionally, we found that the abundances of *Prevotella*, *Gemmiger*, and *Roseburia* in the guts of LC patients were remarkably increased compared with healthy volunteers, consistent with Liu et al.,^10^ Cheng et al.,^39^ and Zhang et al.^40^ This shows that the gut microbiome of LC patients changed dramatically. *Prevotella* belonging to the Prevotellaceae family was found to be associated with immune responses and chronic inflammatory diseases, such as severe asthma,^41^, ^42^ rheumatoid arthritis,^43^ and carboplatin‐induced gut toxicity.^44^ The genus *Gemmiger* was reportedly associated with a decreased risk of *Clostridioides difficile* acquisition^45^ and was enriched in early hepatocellular carcinoma.^39^ The role of *Gemmiger* in the development of LC remains to be further explored in a larger sample cohort. However, Gui et al. also reported that the gut butyrate‐producing bacteria, *Roseburia*, were significantly decreased in NSCLC patients and may affect lung cancer progression and prognosis, which contributed to inhibition of harmful gastrointestinal bacteria growth and intestinal homeostasis by improving the intestinal barrier function.^46^ Therefore, *Prevotella*, *Gemmiger*, and *Roseburia* may serve as a possible diagnostic, prognostic, or therapeutic target in LC therapy. Meanwhile, the KEGG pathway analyses indicated that the dysbiosis of gut bacteria in LC is significantly related to thiamine metabolism. Therefore, we speculated that gut microbiota influences the host via the metabolites, impacting immunologic balance in LC and in which we may identify some promising diagnostic and therapeutic biomarkers.

Metabonomics is becoming one of the most active research areas in the biomedical field, including in cancers.^47^ With the integration of metabolomics in serum and tumour tissue samples, we identified multiple differential metabolites in patients with NSCLC, suggesting that LC pathogenesis may involve an extensive metabolic disturbance. In serum samples, the prominent metabolites were mostly associated with aspartate, alanine, and glutamate metabolism. In tissue samples, the outstanding metabolites were primarily related to GABAergic synapse, intestinal immune network for IgA production, glutamatergic synapse, small cell lung cancer, and so forth. Additionally, intersected metabolic pathways of differential metabolites in serum‐/tissues‐based metabolomics were closely related to arginine, proline, retinol, and caffeine metabolism. Amino acid metabolism is one of the basic metabolic pathways in cancer growth,^48^ and a study has clarified that amino acid metabolism was significantly altered in the T790M‐mutant NSCLC.^49^ Retinol (vitamin A), a fat‐soluble nutrient obtained from diet, can affect cell differentiation, proliferation, and apoptosis‐related BPs.^50^ Caffeine (1,3,7‐trimethylxanthine) is a xanthine alkaloid found in a wide variety of dietary products. But its role in cancer cells is controversial. Xu et al. reported that patients receiving taxanes treatment should avoid consuming caffeinated beverages or foods,^51^ whereas others found caffeine heightened chemo‐sensitivity in NSCLC,^52^ breast cancer,^53^ and prostate cancer cell lines.^54^ Our results suggest that caffeine metabolism is associated with the initiation and progression of NSCLC. However, these results should be verified in randomized clinical trials due to the observational nature, bias and confounding of studies. In addition, the metabolites, including 9(S)‐HPODE, xanthine, and all‐trans‐retinoic acid, were synchronously observed in serum‐/tissue‐based metabolomics with high diagnostic validity. On the other side, the levels of l‐glutamic acid, l‐glutamine, and gamma‐glutamylcysteine were reversed between serum and tumour tissue samples. Glutamine, a multifunctional amino acid, was used as an essential nutrient to support cell growth and proliferation in many types of cancers.^55^ Studies have revealed that the tumour cells consume excess glutamine in vivo, which has also resulted in a decrease of glutamine in blood circulation.^56^, ^57^ Consistent with the previous studies, our study also suggests that glutamine blockade within the solid tumour microenvironment might serve as a candidate therapeutic strategy for LC.

It has previously been reported that the all‐trans retinoic acid attenuates cancer resistance of gefitinib in NSCLC.^58^ Therefore, it is important to investigate the effects of all‐trans retinoic acid, amino acids, and xanthine in LC. To further augment our understanding of the relationship between metabolites and microbiota, a correlation analysis was performed. Spearman's correlation analysis revealed that there was a possible link between *Prevotella* and nervonic acid and all‐trans retinoic acid in LC patients. However, the mode of action of *Prevotella* on nervonic acid and all‐trans retinoic acid is not yet well understood in LC.

The proteomics alterations might help explore the underlying pathological mechanisms of LC.^59^ We also performed an LC/MS‐based proteomics analysis to identify the DEPs of serum in LC patients. It was found that most of the DEPs were mainly involved in the regulation of IL‐8 production, TLR4 signalling pathway, positive regulation of TNF production, and lipopolysaccharide receptor complex. For this study, we selected CRP, LBP, and CD14, which are related to IL‐8 production. A recent study described that CRP could improve the early diagnostic sensitivity of NSCLC,^60^ and CRP was also significantly changed in our study. LBP is produced by lung parenchyma, and Chalubinska‐Fendler et al. have proposed that LBP may be a marker for evaluating radiotoxicity in NSCLC patients.^61^ CD14, a well‐known glycosylphosphatidyl inositol–anchored co‐receptor for TLRs, is dramatically associated with the TLR4‐dependent inflammatory signalling pathways in macrophages.^62^ To this end, proteomics and metabolomics were combined to highlight the connection between the DEPs and metabolites at a higher level. The results showed that CRP, LBP, and CD14 were inversely related to nervonic acid and all‐trans retinoic acid.

In this study, we found that gut microbiota from patients with NSCLC enriched with *P. copri* modulates chronic inflammation and immune dysfunction in the LLC‐bearing mice. To investigate the possible preventive effects of nervonic acid and all‐trans retinoic acid on xenotransplanted tumours in *P. copri*‐treated LLC‐bearing mice, nervonic acid and all‐trans retinoic acid administration was initiated at the same time as *P. copri* gavage to mice. Nervonic acid and all‐trans retinoic acid treatment attenuated the inflammatory reaction, the abnormal lung morphology, and corrected the immune disorder induced by *P. copri*. These data indicate that nervonic acid and all‐trans retinoic acid administration improved inflammatory reaction and immunologic function in the *P. copri*‐treated LLC‐bearing mice. Thus, we demonstrated that the regulation of *Prevotella*, nervonic acid, all‐trans retinoic acid, CRP, LBP, and CD14 could be an underlying therapeutic strategy for the inflammatory treatment of NSCLC.

## 5 CONCLUSION

In summary, our results suggested that both the abnormalities of gut microbiota, metabolism, and proteomics were closely linked with the occurrence and development of NSCLC. We further discovered the vital role of intestinal flora in the progression of NSCLC and a possible relationship among *P. copri*, nervonic acid, all‐trans retinoic acid, CRP, LBP, and CD14 in NSCLC pathogenesis, providing the basis for future studies on the pathogenesis and treatment of NSCLC. However, the current studies are still limited. A larger sample cohort experiment is needed in the future to identify an ideal biomarker for lung cancer. The serum‐ and tissue‐based metabonomics analyses were conducted in different batches of lung cancer patients, leading to variable results. Furthermore, we only revealed a preliminary correlation among lung cancer intestinal microbiota, metabolites, and proteomics, but the causal relationship among *P. copri*‐nervonic acid and all‐trans‐retinoic acid‐CRP, LBP, and CD14 axis needs further investigations.

## CONFLICT OF INTEREST

The authors declare that there is no conflict of interest that could be perceived as prejudicing the impartiality of the research reported.

## Supporting information

Supporting Information.
**Additional file 1**: **Figure S1** **. The relative abundance of gut microbiota in human adults with LC and healthy controls**. (A) Venn diagram depicting shared and unique OTUs for LC and control groups at the phylum and genus levels calculated through the R software. (B) Taxonomic distributions of bacteria at the phylum level and genus level in the control and LC groups. (C) Correlations of 30 significantly discriminant taxa in LC and healthy control patients using Spearman's correlation analysis. Colour and intensity represent the strength of the correlation between bacterial taxa. **Figure S2** **. LDA (linear discriminant analysis) value distribution histogram between LC and control groups**. (A) The cladogram shows differential colonic microbial taxa among the LC and control group. (B) LDA effect size analysis. Histogram of the LDA scores for different abundant genera in healthy control and LC patients. Red, enriched in healthy controls. (C) Functional predictive analysis of gut microbiota in LC patients and healthy controls. Microbial community functions against KEGG database between the LC and control groups predicted by PICRUSt. **Figure S3** **. PCA score plot and relative standard deviation (RSD) of QC samples in serum‐based metabolomics analysis with positive (A) and negative (B) modes**. **Figure S4** **. Representative total ion chromatograms (TIC) of serum samples in positive ion mode (A) and negative ion mode (B) with identified differential metabolites**. **Figure S5** **. Thirteen representative differential metabolites of serum samples are shown using a box map**. Con group versus LC group, *p < .05, **p < .01, ***p < .001. **Figure S6** **. Metabolic pathway analysis of differential metabolites of serum samples between the LC patients and healthy controls**. (a: Alanine, aspartate, and glutamate metabolism; b: arginine and proline metabolism; c: retinol metabolism; d: caffeine metabolism; e: d‐glutamine and d‐glutamate metabolism; f: glutathione metabolism). **Figure S7**. PCA score plot and relative standard deviation (RSD) of QC samples in tumour tissue‐based metabolomics analysis with positive (A) and negative (B) modes. **Figure S8**. Representative total ion chromatograms (TIC) of tumour tissue samples in positive ion mode (A) and negative ion mode (B) with identified differential metabolites. **Figure S9**. Seventeen representative differential metabolites of tumour samples are shown using a box map. T: tumour, N: normal. N group versus T group, *p < .05, **p < .01, ***p < .001. **Figure S10**. Metabolic pathway analysis of differential metabolites of tumour tissue samples between the LC patients and healthy controls (a: GABAergic synapse; b: intestinal immune network for IgA production; c: glutamatergic synapse; d: small cell lung cancer; e: glutathione metabolism; f: nicotine addiction; g: African trypanosomiasis). **Figure S11**. Levels of 9(S)‐HPODE, l‐glutamic acid, xanthine, l‐glutamine, all‐trans‐retinoic acid, and gamma‐glutamylcysteine in serum‐based metabolomics analysis (A) and tissue‐based metabolomics analysis (B). **Figure S12** **. Heat map of correlation analysis between serum metabolites and gut bacteria at the genus level**. In the heat map, colour intensity represents the magnitude of correlation. Red, positive correlation (*r* > .3); blue, negative correlation (*r* < −.3). Significant correlations marked by asterisks (*p < .05, **p < .01, ***p < .001). **Figure S13** **. Heat map of correlation analysis between serum metabolites and differentially expressed proteins**. The abscissa represents the identified DEPs and the ordinate represents serum biomarkers. The colour spectrum from blue to red indicates increasing correlation (*p < .05, **p < .01, ***p < .001).

Supporting Information.
**Additional file 2**: **Table S1**. Clinical features of NSCLC patients. **Table S2**. Data teleprocessing statistics and quality control information of 16S DNA sequencing. **Table S3**. Bacterial libraries after taxonomic assignment of OTUs at 97% minimum similarity level. **Tables S4–S8** show gut microbial community features of LC patients at various levels: **Table S4**. phylum level; **Table S5**. class level; **Table S6**. order level; **Table S7**. family level; **Table S8**. genus level. **Table S9**. Differential metabolites in serum samples between the LC patients and healthy controls. **Table S10**. Differential metabolites in tumour samples between the LC patients and healthy controls. **Table S11**. Intersected metabolic pathways of differential metabolites in serum‐/tissues‐based metabolomics. **Table S12**. ROC curves of diagnostic performance for each metabolite in tissue‐based metabolomics. **Table S13**. Proteins in the serum‐based proteomics analysis. **Table S14**. Summary of differentially expressed proteins in serum samples of LC and healthy controls.
