# Supplementary material for: Integrated microbiome, metabolome, and proteome analysis identifies a novel interplay among commensal bacteria, metabolites and candidate targets in non‐small cell lung cancer
Source: Clin Transl Med. 2022 Jun 23;12(6):e947. doi: 10.1002/ctm2.947 (PMC9218934; doi:10.1002/ctm2.947)
Supplement: Supplementary file 1 — Supporting Information. Additional file 1: Figure S1 . The relative abundance of gut microbiota in human adults with LC and healthy controls. (A) Venn diagram depicting shared and unique OTUs for LC and control groups at the phylum and genus levels calculated through the R software. (B) Taxonomic distributions of bacteria at the phylum level and genus level in the control and LC groups. (C) Correlations of 30 significantly discriminant taxa in LC and healthy control patients using Spearman's correlation analysis. Colour and intensity represent the strength of the correlation between bacterial taxa. Figure S2 . LDA (linear discriminant analysis) value distribution histogram between LC and control groups. (A) The cladogram shows differential colonic microbial taxa among the LC and control group. (B) LDA effect size analysis. Histogram of the LDA scores for different abundant genera in healthy control and LC patients. Red, enriched in healthy controls. (C) Functional predictive analysis of gut microbiota in LC patients and healthy controls. Microbial community functions against KEGG database between the LC and control groups predicted by PICRUSt. Figure S3 . PCA score plot and relative standard deviation (RSD) of QC samples in serum‐based metabolomics analysis with positive (A) and negative (B) modes. Figure S4 . Representative total ion chromatograms (TIC) of serum samples in positive ion mode (A) and negative ion mode (B) with identified differential metabolites. Figure S5 . Thirteen representative differential metabolites of serum samples are shown using a box map. Con group versus LC group, *p < .05, **p < .01, ***p < .001. Figure S6 . Metabolic pathway analysis of differential metabolites of serum samples between the LC patients and healthy controls. (a: Alanine, aspartate, and glutamate metabolism; b: arginine and proline metabolism; c: retinol metabolism; d: caffeine metabolism; e: d‐glutamine and d‐glutamate metabolism; f: glutathione metabolism). Figure S7. PC [file CTM2-12-e947-s001.doc]

**Supplementary Figure legends**

**
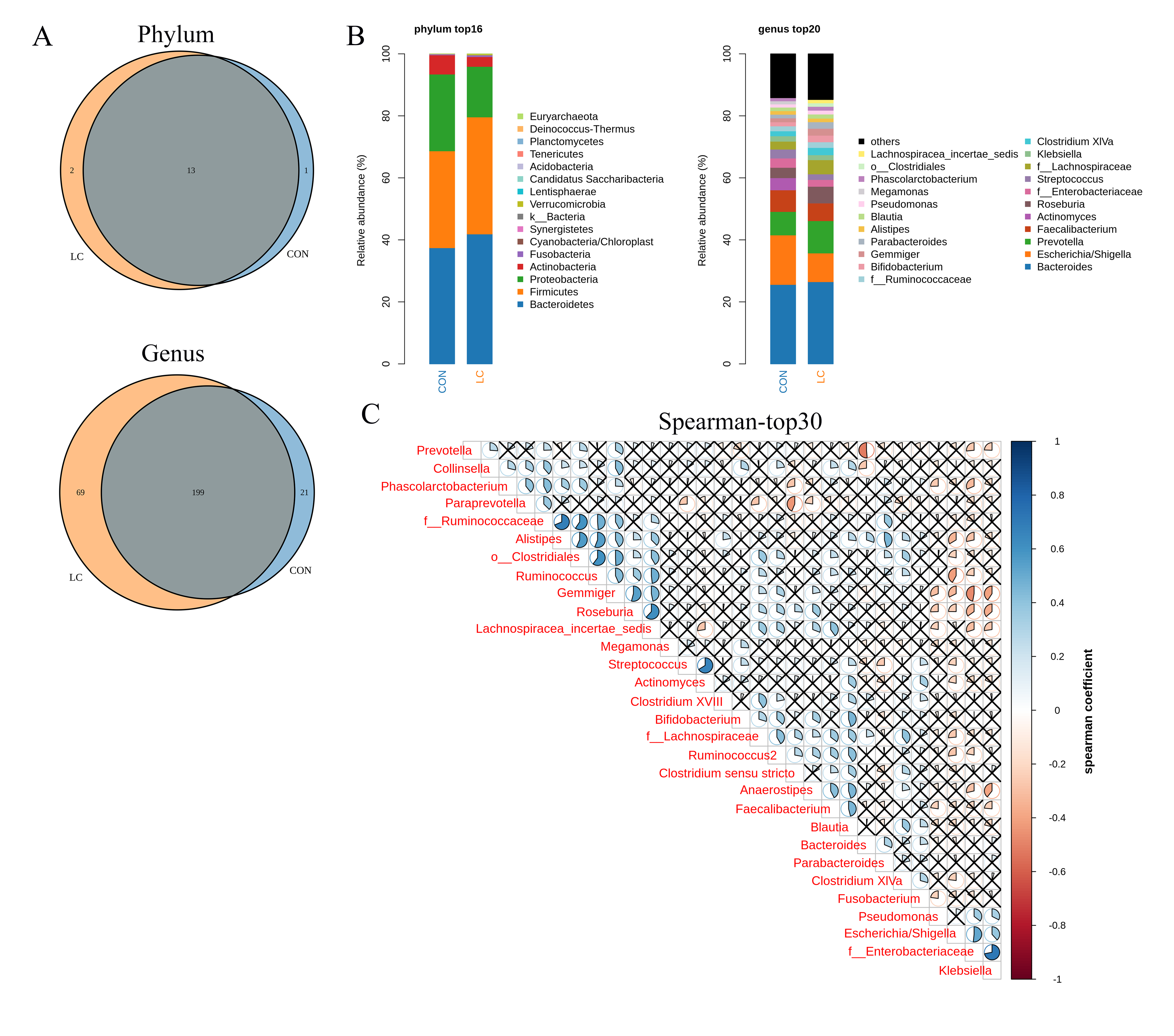
**

**Figure S1. The relative abundance of gut microbiota in human adults with LC and healthy controls**. (A) Venn diagram depicting shared and unique OTUs for LC and control groups at the phylum and genus levels calculated through the R software. (B) Taxonomic distributions of bacteria at the phylum level and genus level in the control and LC groups. (C) Correlations of 30 significantly discriminant taxa in LC and healthy control patients using Spearman correlation analysis. Color and intensity represent the strength of the correlation between bacterial taxa.

**
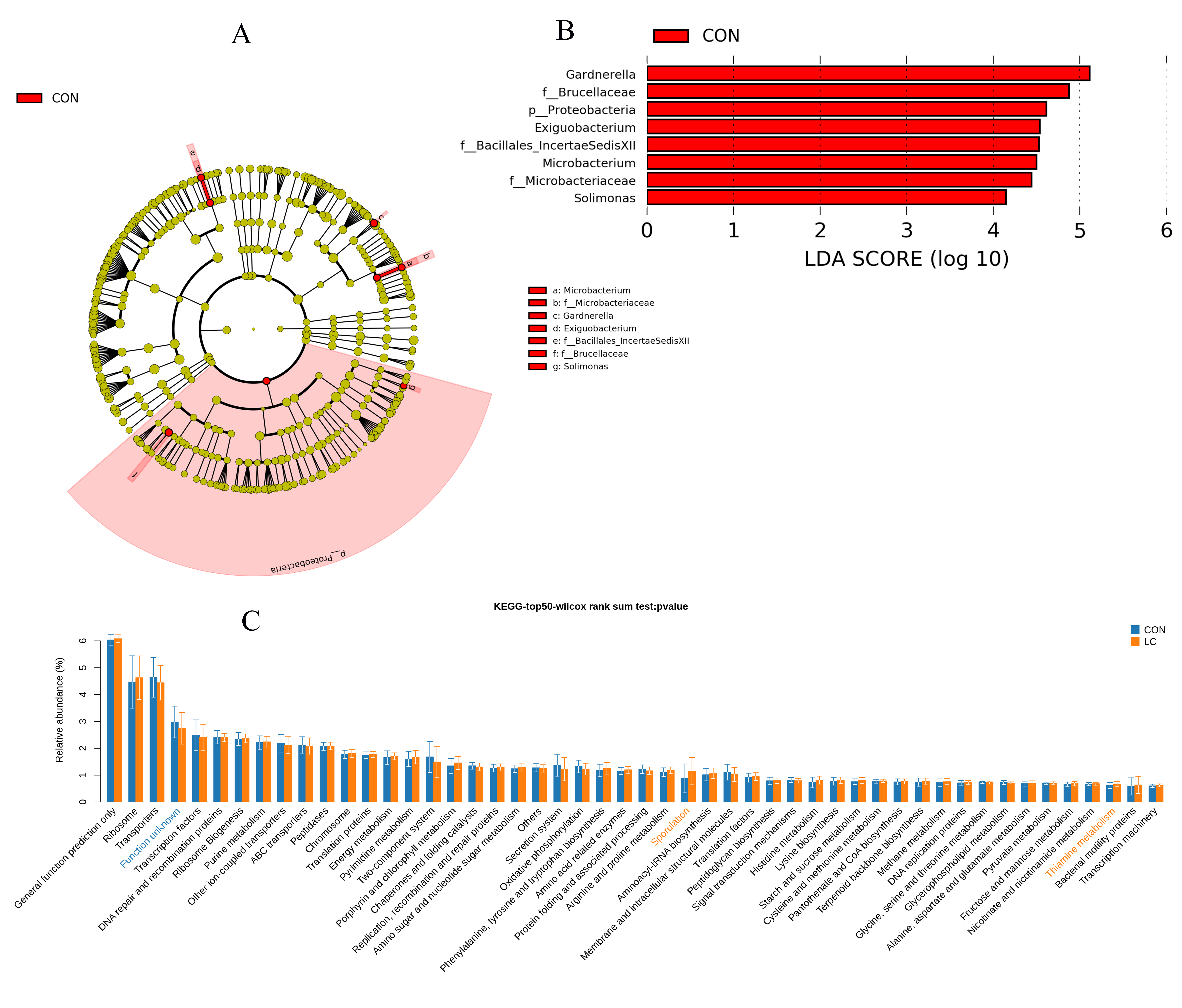
**

**Figure S2. LDA (Linear discriminant analysis) value distribution histogram between LC and control groups** (A) The cladogram shows differential colonic microbial taxa among the LC and control group. (B) LDA effect size analysis. Histogram of the LDA scores for different abundant genera in healthy control and LC patients. Red, enriched in healthy controls. (C) Functional predictive analysis of gut microbiota in LC patients and healthy controls. Microbial community functions against KEGG database between the LC and control groups predicted by PICRUSt.


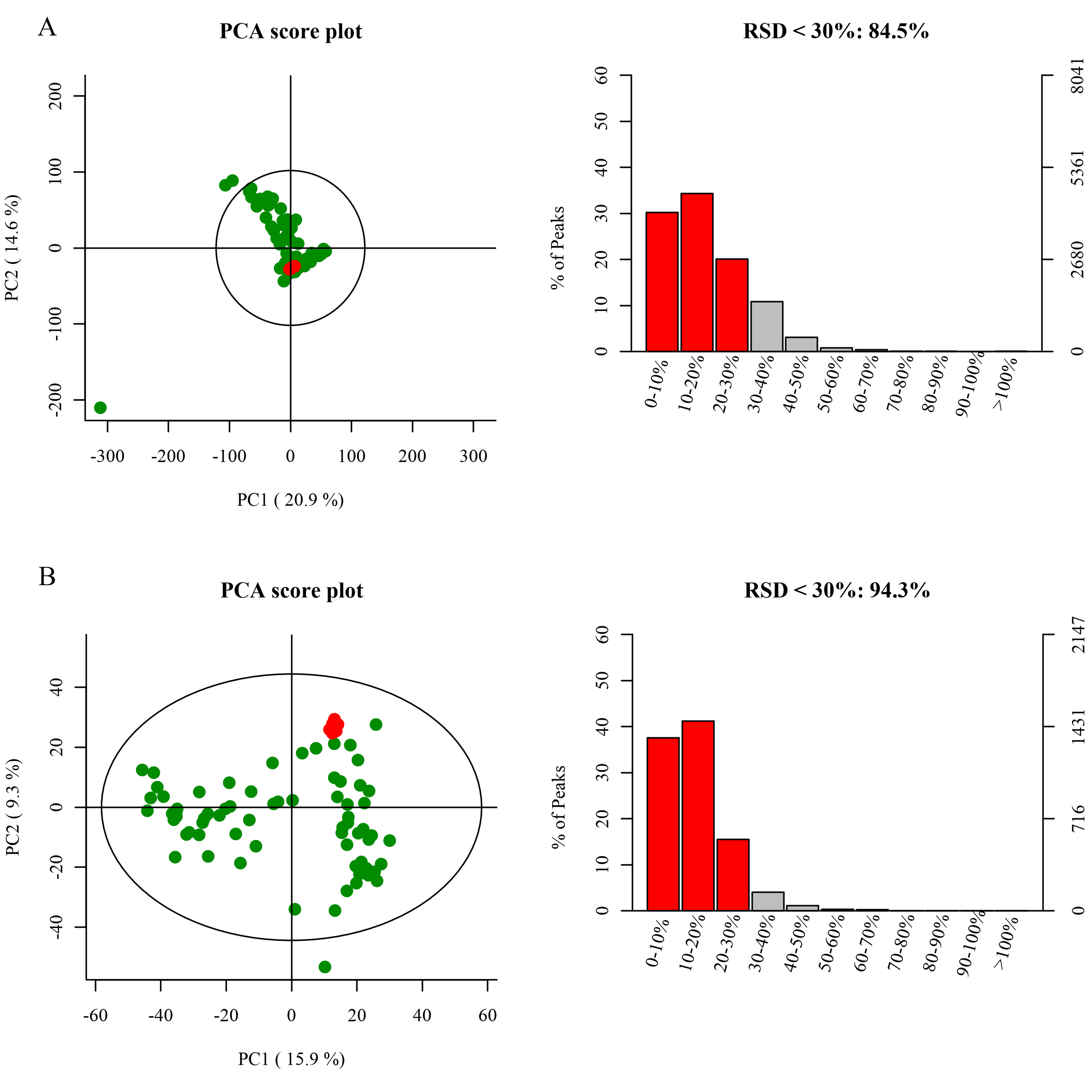


**Figure S3 PCA score plot and Relative standard deviation (RSD) of QC samples in serum-based metabolomics analysis with positive (A) and negative (B) modes**.

**
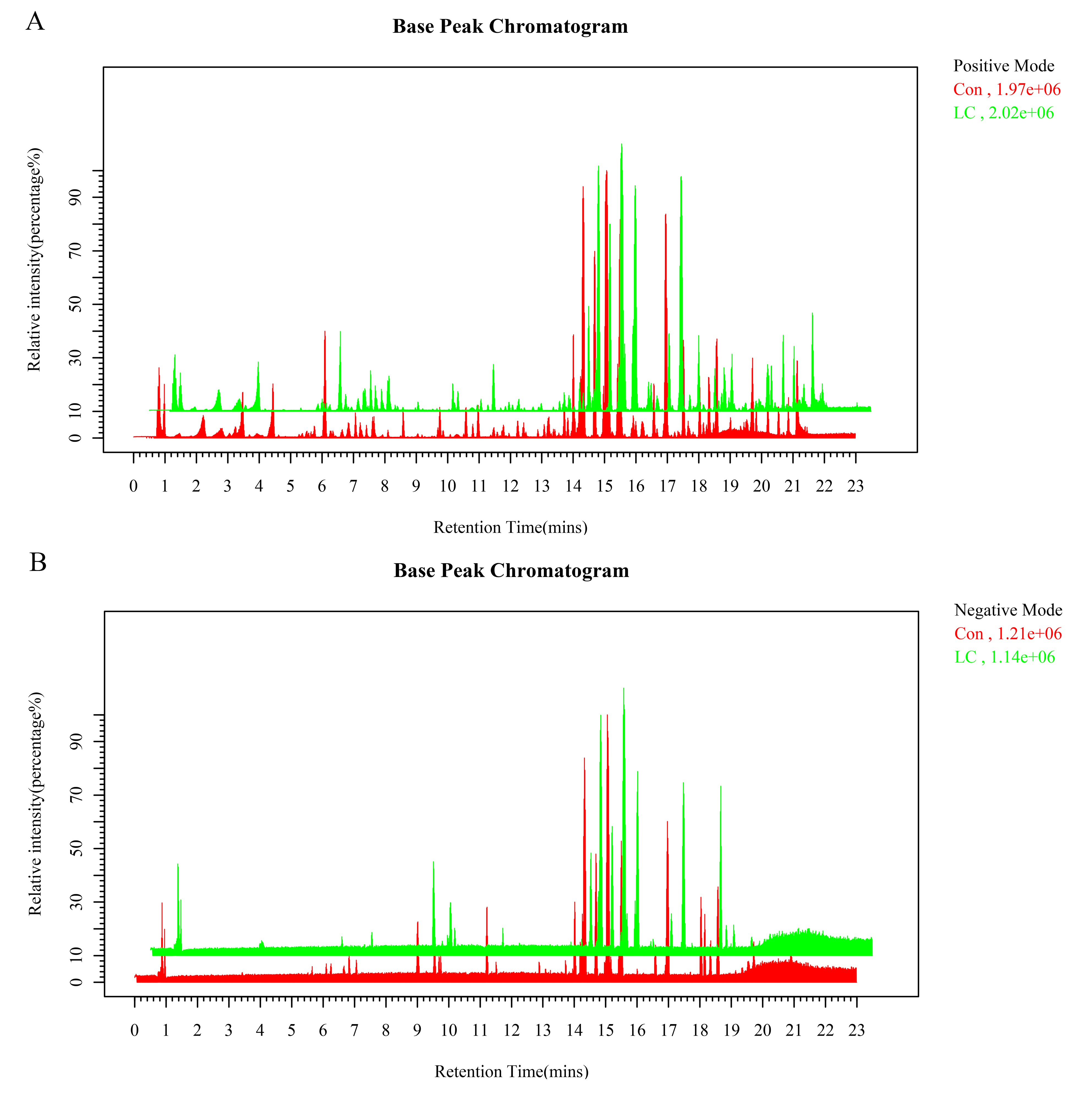
**

**Figure S4** **Representative total ion chromatograms (TIC) of serum samples in positive ion mode (A) and negative ion mode (B) with identified differential metabolites**.





**Figure S5 Thirteen representative differential metabolites of serum samples are shown using a box map**. Con group *vs.* LC group, **P < 0.05*, ***P < 0.01*, ****P < 0.001.*

**
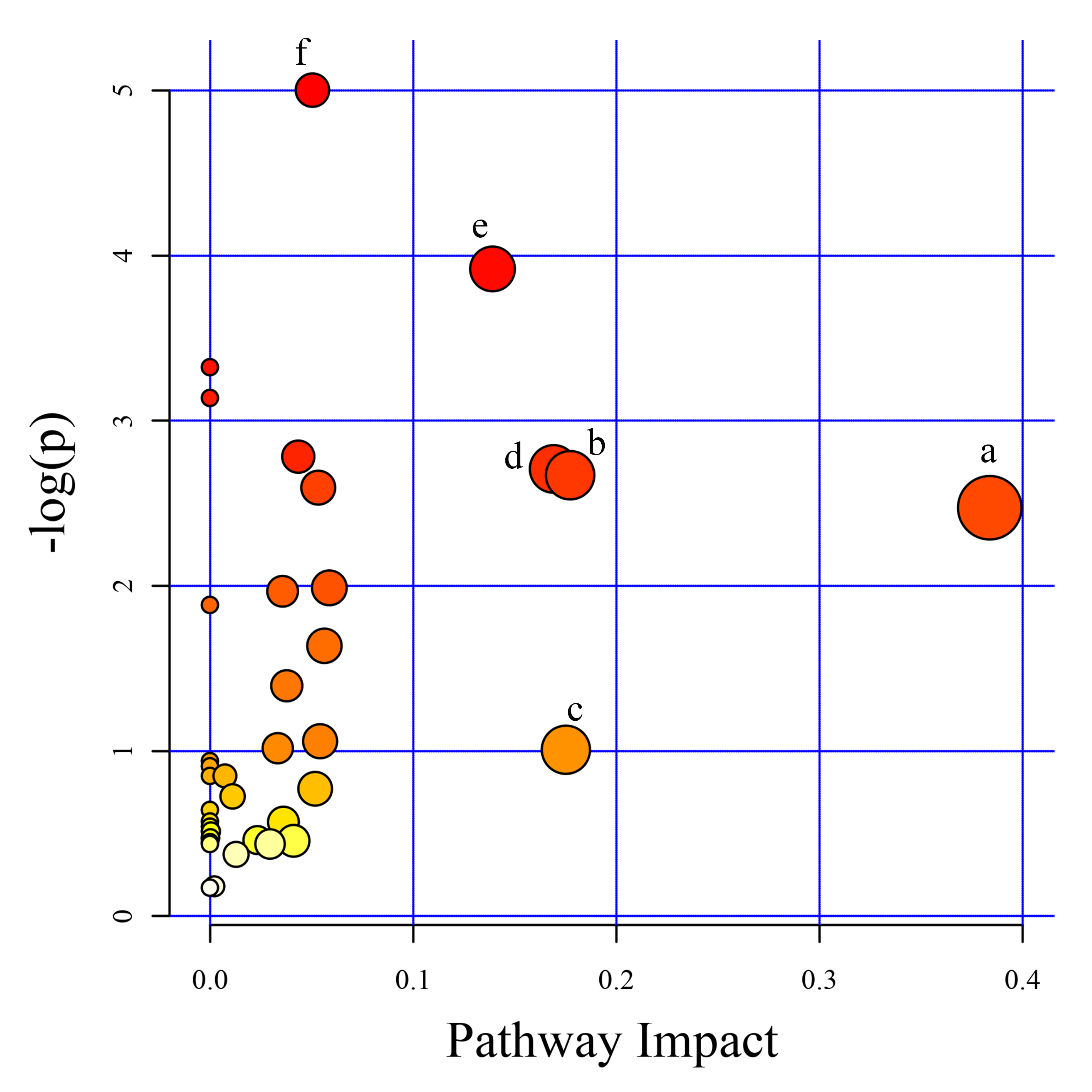
**

**Figure S6** **Metabolic pathway analysis of differential metabolites of serum samples between the LC patients and healthy controls.** (a: Alanine, aspartate and glutamate metabolism; b: Arginine and proline metabolism; c: Retinol metabolism; d: Caffeine metabolism; e: D-Glutamine and D-glutamate metabolism; f: Glutathione metabolism).

**
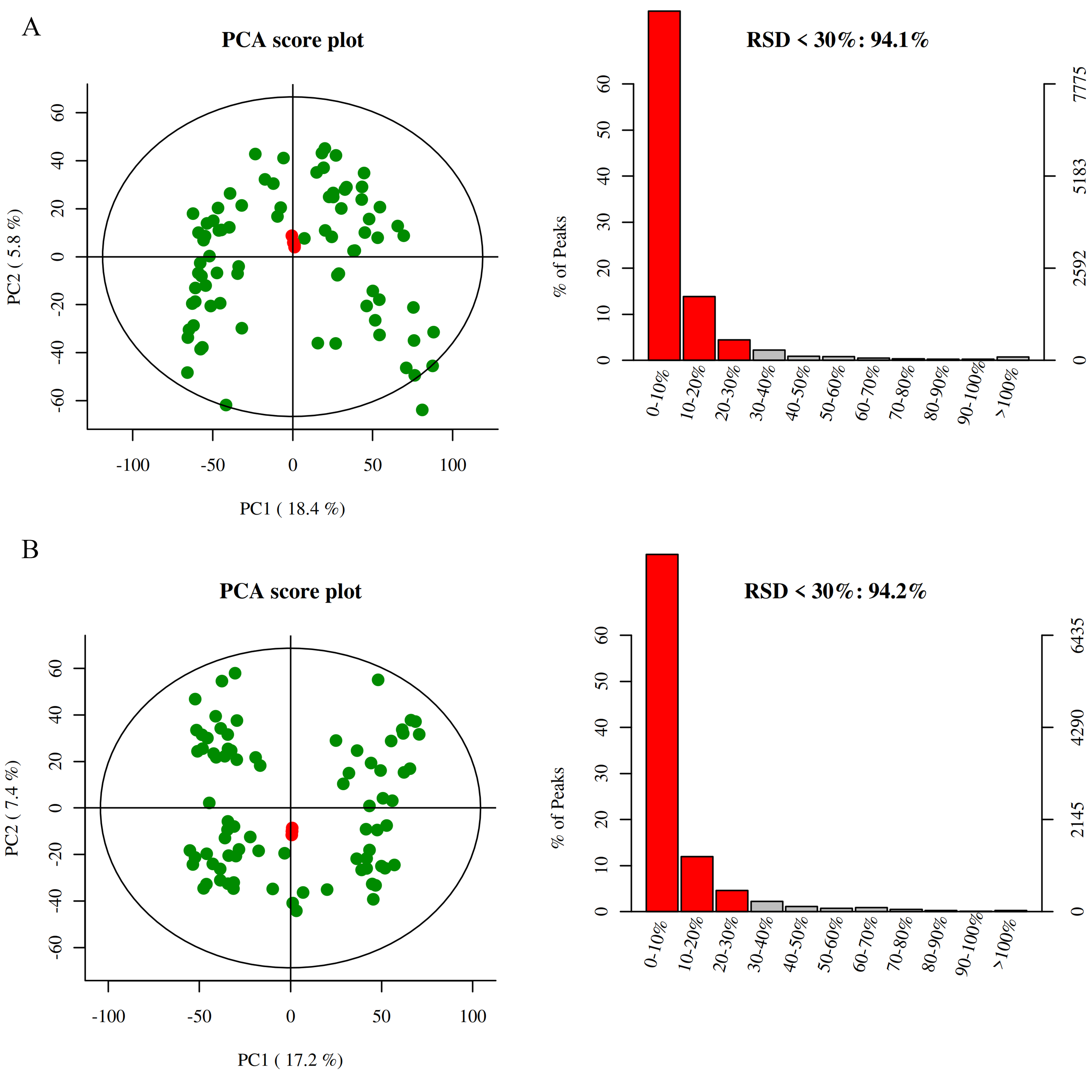
**

**Figure S7** PCA score plot and Relative standard deviation (RSD) of QC samples in tumor tissue-based metabolomics analysis with positive (A) and negative (B) modes.

**
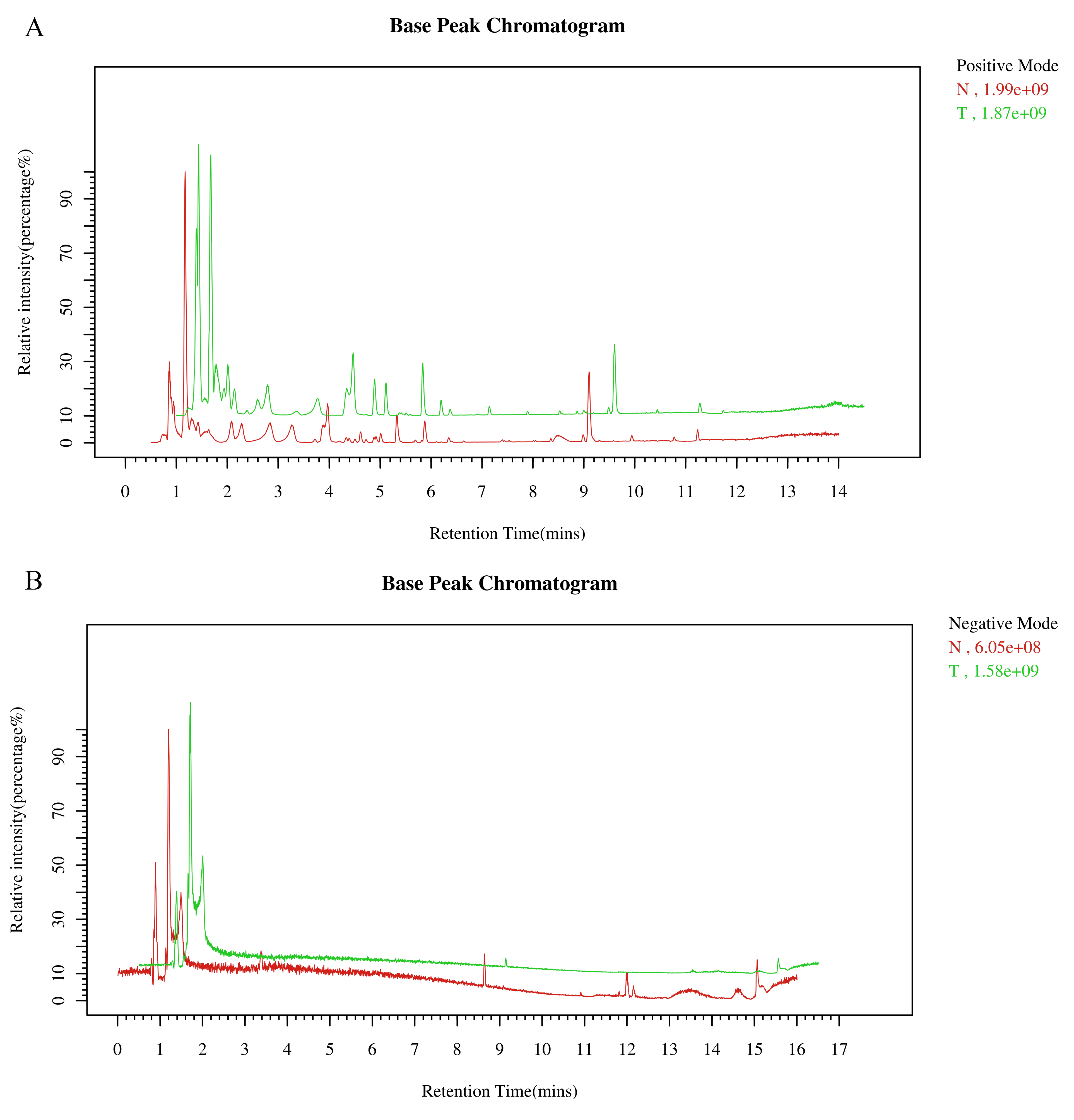
**

**Figure S8** Representative total ion chromatograms (TIC) of tumor tissue samples in positive ion mode (A) and negative ion mode (B) with identified differential metabolites.


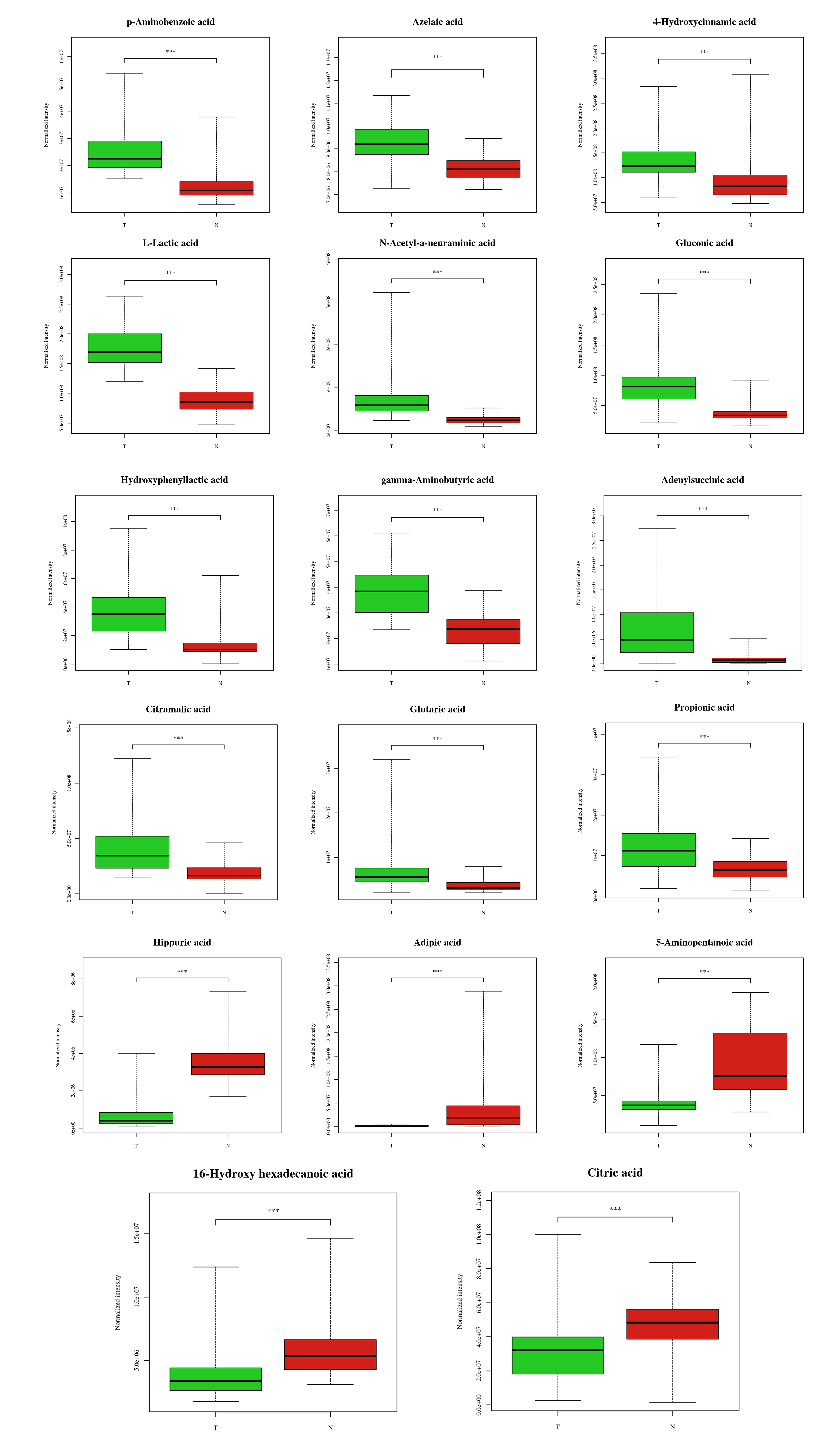


**Figure S9** Seventeen representative differential metabolites of tumor samples are shown using a box map. T: tumor, N: normal. N group *vs.* T group, **P < 0.05*, ***P < 0.01*, ****P < 0.001.*

**
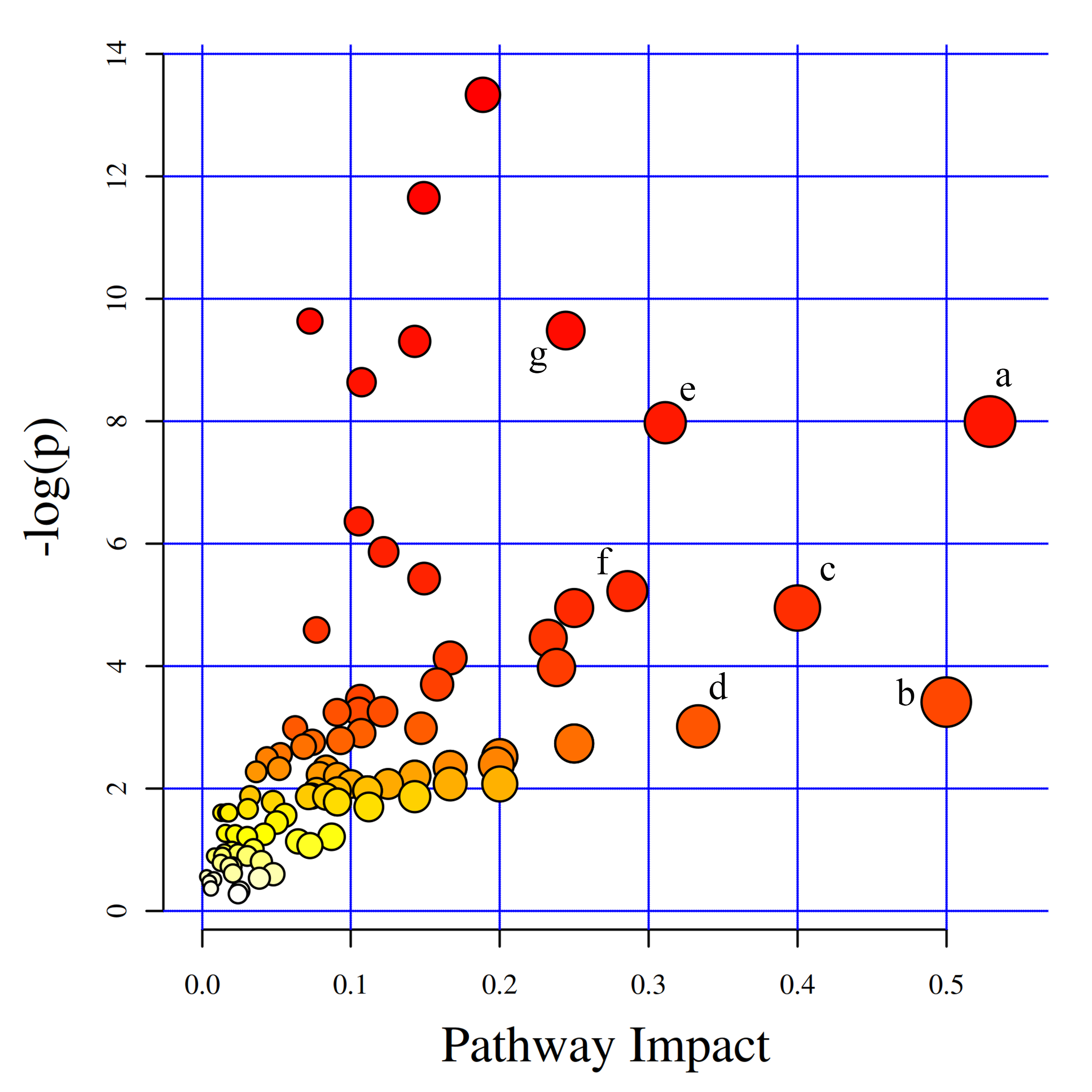
**

**Figure S10** Metabolic pathway analysis of differential metabolites of tumor tissue samples between the LC patients and healthy controls (a: GABAergic synapse; b: Intestinal immune network for IgA production; c: Glutamatergic synapse; d: Small cell lung cancer; e: Glutathione metabolism; f: Nicotine addiction; g: African trypanosomiasis).





**Figure S11.** Levels of 9(S)-HPODE, L-glutamic acid, xanthine, L-glutamine, all-trans-retinoic acid, and gamma-glutamylcysteine in serum-based metabolomics analysis (A) and tissue-based metabolomics analysis (B).

**

**

**Figure S12. Heat map of correlation analysis between serum metabolites and gut bacteria at the genus level.** In the heat map, color intensity represents the magnitude of correlation. Red, positive correlation (r > 0.3); blue, negative correlation (r < −0.3). Significant correlations marked by asterisks (**P < 0.05*, ***P < 0.01*, ****P < 0.001*).

**
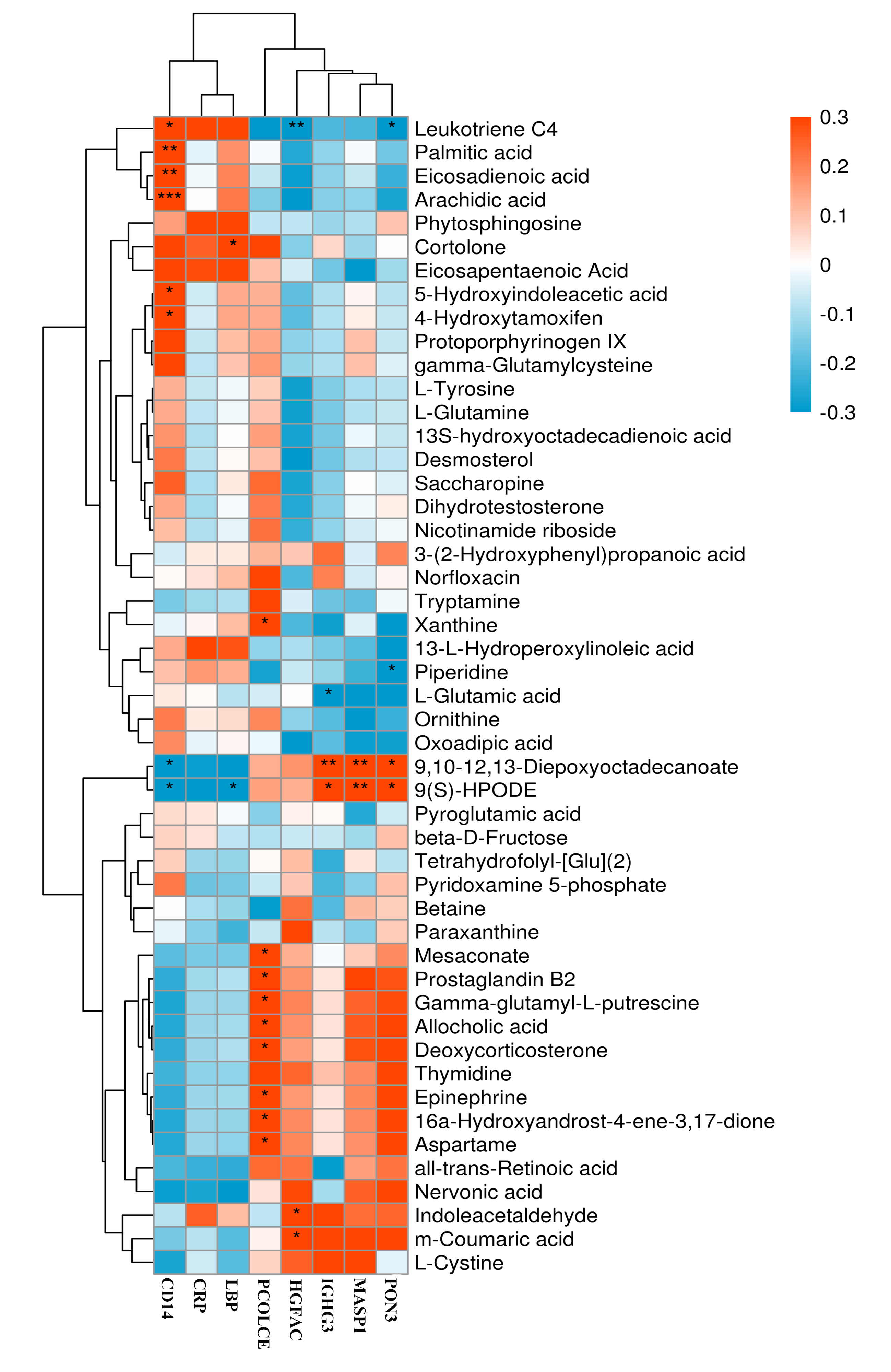
**

**Figure S13. Heat map of correlation analysis between serum metabolites and differentially expressed proteins.** The abscissa represents the identified DEPs and the ordinate represents serum biomarkers. The color spectrum from blue to red indicates increasing correlation (**P < 0.05*, ***P < 0.01*, ****P < 0.001*).
